# Exploring Daptomycin Hypersensitivity in *Enterococcus faecium*: The Impact of LafB Mutation on Bacterial Virulence

**DOI:** 10.3390/ijms26135935

**Published:** 2025-06-20

**Authors:** Pamela I. Huanambal Esquén, Diego A. Leonardo, Livia R. Manzine, Erick Suclupe Farro, Jessica K. Kajfasz, Suelen S. Mello, Mara C. L. Nogueira, João Renato Muniz, Alessandro S. Nascimento, Michael S. Gilmore, Jacqueline Abranches, José A. Lemos, Ilana L. B. C. Camargo

**Affiliations:** 1 Department of Physics and Interdisciplinary Science, Sao Carlos Institute of Physics, University of Sao Paulo, Sao Carlos 13563-120, Brazil; pamelahuanambal@gmail.com (P.I.H.E.); dleonardo@usp.br (D.A.L.); livia@ifsc.usp.br (L.R.M.); esuclupef@ifsc.usp.br (E.S.F.); jrcmuniz@ifsc.usp.br (J.R.M.); asnascimento@ifsc.usp.br (A.S.N.); 2 Department of Oral Biology, University of Florida College of Dentistry, Gainsville, FL 32610, USA; jkajfasz@dental.ufl.edu (J.K.K.); jabranches@dental.ufl.edu (J.A.); jlemos@dental.ufl.edu (J.A.L.); 3 Department of Ophthalmology, Harvard Medical School, Boston, MA 02114, USA; suelen_scarpademello@meei.harvard.edu (S.S.M.); michael_gilmore@meei.harvard.edu (M.S.G.); 4 Departamento de Doenças Dermatológicas, Infecciosas e Parasitárias, Faculdade de Medicina de São José do Rio Preto—FAMERP, São José do Rio Preto 15090-000, Brazil; ml.nogueira@famerp.br

**Keywords:** LafB, daptomycin hypersusceptibility, *Enterococcus faecium*, VRE

## Abstract

Daptomycin (DAP) is a therapeutic option for vancomycin-resistant *Enterococcus faecium* (VRE) infections, but DAP resistance may occur during treatment. Previously, we identified a mutation within the *E. faecium lafB* gene that induces hypersusceptibility to DAP. The *lafB* gene encodes a glycosyltransferase involved in lipoteichoic acid anchor synthesis, which makes it a promising target for enhancing DAP efficacy. In this study, we characterized *E. faecium* LafB protein (*Ef*LafB) biophysical properties, used AlphaFold3 to predict LafB in silico three-dimensional structure, and determined *lafB* gene mutation’s role in virulence, comparing *E. faecium* HBSJRP18 (DAP-hypersusceptible) and a *lafB* revertant, HBSJRP18_2.7, and analyzing bacterial growth kinetics, biofilm formation ability, and virulence in a *Galleria mellonella* model. After gene cloning and expressing and purifying *Ef*LafB, circular dichroism and SEC-MALS assays revealed its monomeric nature under in vitro conditions, with approximately a 40 kDa molecular mass and a melting temperature of 50 °C. In silico prediction indicated that LafB is an αβ-type protein with two domains conforming to the GT-4 family glycosyltransferases. These results are further supported by the highly conserved amino acids (E257, D91, R184, and K185), likely involved in UDP-Glc binding. The studied *lafB* gene mutation resulted in a significant decrease in bacterial growth and virulence in the invertebrate model.

## 1. Introduction

The WHO designated vancomycin-resistant *Enterococcus faecium* (VREfm) as a priority pathogen, prompting the development of new antibiotics [1]. While most vancomycin-resistant *Enterococcus* (VRE) strains remain susceptible to last-resort antibiotics, such as linezolid, tigecycline, and daptomycin (DAP), in vitro, DAP has proven bactericidal activity only at high dosages licensed for treating enterococcal endocarditis [2]. Linezolid is generally recommended as an alternative treatment for VRE-induced endocarditis when no other alternative is available. However, resistance to these antibiotics has already been reported [3] in *Enterococcus* spp., which are clinically important in hospitals and healthy animals, including plasmid-mediated linezolid resistance mechanisms [3,4,5,6,7,8,9,10]. Therefore, there is an urgent need for new compounds to combat VREfm infection.

DAP, a cyclic lipopeptide antibiotic, is a possible alternative treatment for various bacterial infections caused by Gram-positive bacteria, including methicillin-resistant *Staphylococcus aureus* (MRSA) and vancomycin-resistant enterococci (VRE), depending on the infection site. FDA-approved clinical uses of DAP include complicated skin and skin structure infections (cSSSIs) in both adult and pediatric patients, *S. aureus* bacteremia in adults (including those with right-sided infective endocarditis), and *S. aureus* bacteremia in pediatric patients aged 1–17 years. In addition, the off-label clinical use of DAP encompasses diabetic foot infections, cerebrospinal fluid shunt infection, left-sided infective endocarditis caused by *S. aureus* or *Enterococcus* spp. in adults, osteomyelitis and septic arthritis due to methicillin-resistant *Staphylococcus aureus* (MRSA), native vertebral osteomyelitis, intracranial or spinal epidural abscess, prosthetic joint infections caused by Staphylococci or Enterococci, septic arthritis, and VRE infections [11].

The breakpoint for susceptible-dose dependence, defined by the Clinical Laboratory Standard Institute [12], is based on a dosage regimen of 8–12 mg/kg administered every 24 h and intended for serious infections caused by *E. faecium*. Conversely, the daptomycin dosing regimens endorsed by the European Medicines Agency (EMA) consist of lower doses than those in the United States (4–6 mg/kg/day), primarily designed for *Staphylococcus aureus* infections, resulting in insufficient drug exposure for treating enterococcal bloodstream infections (BSIs) [13,14,15]. Studies by the European Committee on Antimicrobial Susceptibility Testing (EUCAST) have indicated that even with doses ranging from 10 to 12 mg/kg/day, it remains challenging to effectively treat infections caused by *Enterococcus faecalis* with MICs of 4 mg·L^−1^ and *E. faecium* with MICs of 4 or 8 mg·L^−1^. Therefore, the EUCAST lists daptomycin breakpoints for *Enterococcus* species as having “IE” (insufficient evidence) and advises increased vigilance when considering high-dose DAP for the treatment of enterococcal BSIs and endocarditis [15].

In 2019, our research group identified a daptomycin-hypersusceptible *E. faecium* strain (*E. faecium* HBSJRP18) belonging to ST412, with a DAP MIC of 0.06 mg·L^−1^, substantially lower than the typical DAP MIC for this species (2 mg·L^−1^) [5,16]. The hypersusceptible phenotype of the clinical isolate *E. faecium* HBSJRP18 was due to a mutation in the *lafB* gene (C557T/Arg193Trp, positions of ORF HMPREF0351_10963 of the *E. faecium* DO genome (NC_017960.1)) [5]. The *lafB* gene encodes LafB glycosyltransferase, which is necessary to form the lipoteichoic acid (LTA) anchor. Studies in *E. faecalis* have identified *bgsA* as a homolog of *lafB* [17,18,19].

Through graded selection in DAP, a wild-type *lafB* revertant, *E. faecium* HBSJRP18_2.7, was obtained, exhibiting a typical DAP MIC of 2 mg·L^−1^ [5]. The mutation in the *E. faecium* glycosyltransferase gene *lafB* resulted in daptomycin hypersusceptibility, and targeting LafB may be of value as an adjuvant to DAP therapy, reducing DAP MIC and possibly restoring DAP activity in DAP-resistant strains.

LTA has been discovered in numerous Gram-positive pathogenic bacteria and plays a key role in surface adherence, cell invasion, and biofilm formation. D-alanine residues in LTA are important for adherence on polar and nonpolar surfaces. In our previous study, FTIR analysis identified qualitative differences in LTA extracted from the HBSJRP18 clinical isolate and reverted evolved strains. FTIR showed that the hypersusceptible *lafB* genotype results in a cell envelope with changed content of fatty acids, phospholipids, and glycolipids [5,20,21]. Although our group is still working on this, we have no answer yet on whether the function or activity of the mutated LafB is compromised or not.

However, understanding the structure of LafB would inform the search for new leads with potential value in limiting DAP resistance and, as we hypothesized, in decreasing virulence, too. This study aimed to characterize LafB biophysically, explore its structure in silico using artificial intelligence (AlphaFold3), and assess the impact of the *lafB* mutation on *E. faecium* virulence. We observed that LafB is an αβ-type, 40 kDa, monomeric protein with two domains conforming to the GT-4 family glycosyltransferases, which is less stable when mutated and also plays a role in bacterial virulence.

## 2. Results

In this study, we biophysically characterized the LafB protein from *E. faecium* HBSJRP18 (*Ef*LaB2.1) and *E. faecium* HBSJRP18_2.7 (*Ef*LafB), conducted an in silico prediction of the mutated (*Ef*LafB2.1) and wild-type (*Ef*LafB) proteins, and verified the role of this mutation in virulence.

### 2.1. The W193R Mutation Reduces the Stability of EfLafB

**Figure 1:**
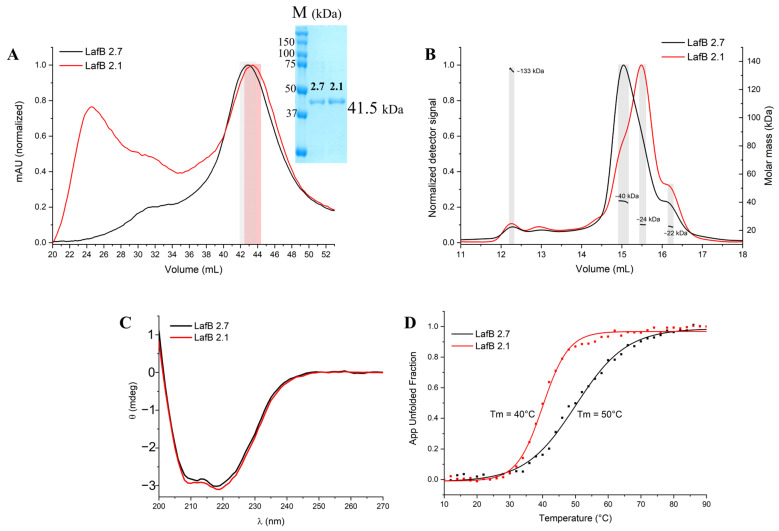
(**A**) Size-exclusion chromatography (SEC) profiles of wild-type (*Ef*LafB, black) and mutant (*Ef*LafB2.1, red) proteins. *Ef*LafB shows a well-defined peak, while *Ef*LafB2.1 exhibits broader, less defined peaks indicative of aggregation. The shaded region highlights the fractions collected for SEC-MALS and CD analyses. (**B**) SEC-MALS results, confirming that *Ef*LafB is monomeric (~40 kDa), whereas *Ef*LafB2.1 shows heterogeneity and potential degradation. (**C**) Circular dichroism (CD) spectra of both proteins, showing characteristic αβ protein folding. (**D**) Thermal denaturation curves of *Ef*LafB proteins, indicating a lower melting temperature (Tm = 40 °C) for *Ef*LafB2.1 compared to *Ef*LafB (Tm = 50 °C), suggesting reduced stability for the mutant. App Unfolded Fraction, Apparent Unfolded Fraction.

The recombinant LafB genes were cloned and expressed as soluble proteins in *E. coli*, which were successfully purified using chromatographic techniques. Size-exclusion chromatography (SEC) showed that the wild-type *Ef*LafB was predominantly homogeneous in solution, whereas the mutant *Ef*LafB2.1 exhibited a significant presence of aggregated protein species, as indicated by broader and less defined elution profiles (Figure 1A).

The fractions highlighted in Figure 1A were collected and further analyzed using size-exclusion chromatography coupled with multi-angle light scattering (SEC-MALS) (Figure 1B). SEC-MALS analysis confirmed that the *Ef*LafB profile maintained a well-defined, homogeneous peak with a calculated molecular mass of approximately 40 kDa, consistent with its theoretical monomeric state. In contrast, *Ef*LafB2.1 exhibited a heterogeneous profile with multiple species of varying molecular weights.

This profile may reflect *Ef*LafB2.1 instability under high-concentration conditions (2 mg·mL^−1^), in contrast to the structural integrity observed at lower concentrations in subsequent CD experiments (0.25 mg·mL^−1^). Moreover, we hypothesized that the observed presence of proteins with lower molecular masses in the SEC-MALS analysis of *Ef*LabB2.1 could be attributed to either spontaneous degradation during concentration or aggregation, which may have led to an increased concentration and thus detection of minor contaminants.

To further investigate the structural properties of both proteins in solution, circular dichroism (CD) spectroscopy was performed. The CD spectra of both *Ef*LafB and *Ef*LafB2.1 revealed characteristic minima at 208 and 222 nm, indicative of an αβ protein fold (Figure 1C). This suggests that the mutation did not drastically alter the secondary structure of the protein at lower concentrations. However, when evaluating thermal stability using CD thermal denaturation assays, significant differences were observed. The melting temperature (Tm) of *Ef*LafB was determined to be approximately 50 °C, whereas the mutant *Ef*LafB2.1 exhibited a lower Tm of 40 °C (Figure 1D). The reduction in Tm by 10 °C suggests that the W193R mutation significantly decreased the thermal stability of *Ef*LafB, making it more susceptible to unfolding at physiological temperatures. Given that SEC-MALS was performed at a higher protein concentration (2 mg·mL^−1^) than CD (0.25 mg·mL^−1^), it is possible that the increased aggregation observed for *Ef*LafB2.1 in SEC-MALS was concentration-dependent, exacerbating the instability of the mutant protein under these conditions.

### 2.2. Sequence and Structural in Silico Analysis

Conservation and similarity analyses of the amino acid sequence, based on the probability of amino acid occurrence at specific positions within aligned sequences using PSI-BLAST [22], identified LafB as a glycosyltransferase of family 4 (GT-4). The GT-4 family is characterized by β-glucosyltransferase folding (GTB-4) and a retaining mechanism [23,24]. A phylogenetic analysis showed that *Ef*LafB is closely related to the GTB-4 family activity group, facilitating the identification of its potential function and substrate specificity. *Ef*LafB showed a closer phylogenetic relationship with the activity group 1,2-diacylglycerol 3-α-glucosyltransferase (Figures S1 and S2), which is involved in the synthesis of glycolipids in the bacterial cell membrane using UDP-glucose (UDP-Glc) or UDP-galactose (UDP-Gal) [18,25].

**Figure 2:**
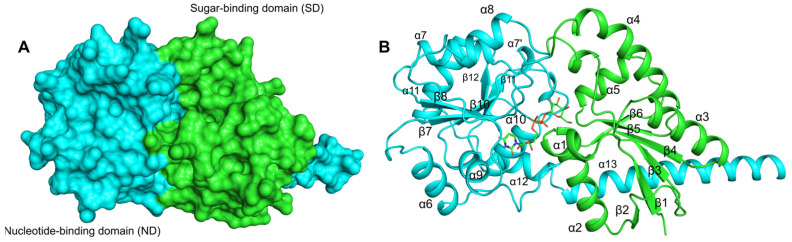
Protein structure model of *Ef*LafB predicted by AlphaFold3. (**A**) The sugar-binding domain (green, in the N-terminus) and the nucleotide-binding domain (cyan, in the C-terminus) show the classic αβα Rossmann fold, each consisting of 6 six β-strands and 6 six α-helices. (**B**) The substrate UDP-Gal is depicted in stick representation, shown bound to the two domains.

The predicted structure of *Ef*LafB showed a high degree of reliability according to the pLDDT (>90) values and the PAE score (<10) (Figure S3). The *Ef*LafB structure presents the classic GTB fold with a sugar-binding domain (SD, green) and a nucleotide-binding domain (ND, cyan) (Figure 2). Both the SD and the ND domains feature a βαβ Rossmann fold type [26,27], consisting of six β-strands and six α-helices. Molecular docking revealed the binding of UDP-Gal between the ND and SD domains with an affinity of −8.2 kcal/mol, indicating a suitable binding interaction. This substrate-binding site has been observed in other crystallographic glycosyltransferases in complex with their ligands [28,29,30].

**Figure 3:**
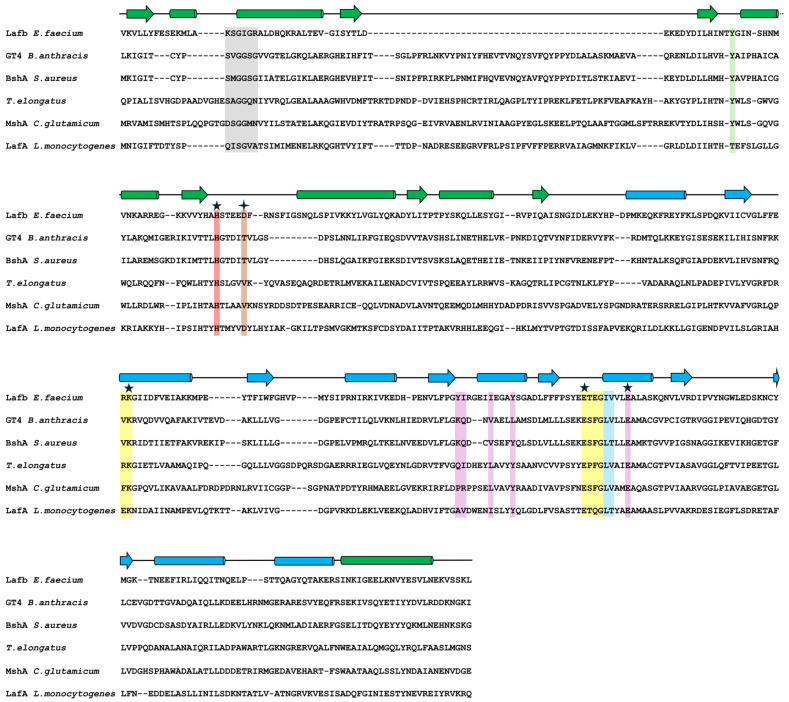
Multiple sequence alignment. Alignment of *Ef*LafB, LafA from *Listeria monocytogenes*, and 4 prokaryotic glycosyltransferases with known structures. The secondary structure elements are shown based on *Ef*LafB, with strands represented as arrows, and helices as cylinders. The sugar-binding domain (SD) is shown in green, and the nucleotide-binding domain (ND) in cyan. Regions interacting with UDP-Gal are highlighted as follows: the glycine-rich region in gray, phosphate interaction regions in light blue, uridine interaction regions in yellow, and sugar interaction regions in violet. Additionally, regions critical for catalytic activity are indicated in red, orange, and light green. Stars indicate residues essential for glycosyltransferase activity.

Multiple sequence alignment (MSA) based on the protein structure of *Ef*LafB and prokaryotic glycosyltransferases with experimentally determined structures (PDB codes: 2JJM, 6N1X, 3C4Q, and 6KIH) [28,29,30,31] validated the interactions between specific residues of *Ef*LafB and the substrate UDP-Gal suggested by molecular docking (Figure 3).

**Figure 4:**
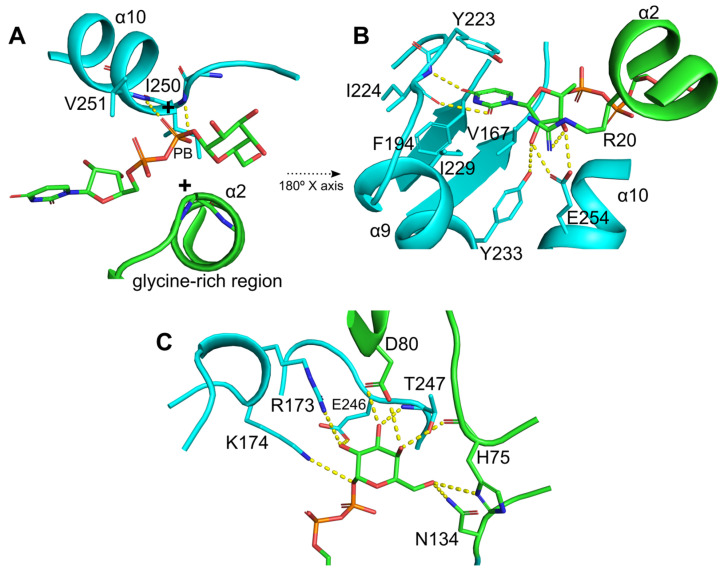
Conserved interaction network and substrate specificity-related residues in UDP-Gal binding. (**A**) The glycine-rich region in the N-terminus of the α2 helix interacts with the nucleotide phosphates. Additionally, the main chain of I250 and V251, located at the N-terminus of the α10 helix, form hydrogen bonds with the β-phosphate (PB). (**B**) Hydrogen bond and hydrophobic interactions between the uridine moiety and residues in the β9-α9 loop and α9 and α10 helices. Among these, E254, a highly conserved residue in glycosyltransferases that binds UDP, plays a key role in specificity. (**C**) Residues involved in UDP-Gal recognition and stabilization of the galactose moiety. The R173/K174 pair stabilizes the negative charge of the phosphate groups after sugar release, analogously to the role of divalent cations in GT-A glycosyltransferases. The highly conserved residue E246 is critical for catalytic activity and sugar specificity, as mutations at this site result in total loss of the enzymatic function. Additionally, SD domain residues such as H75 and Y52 contribute to substrate recognition, while D80, a characteristic residue in *Ef*LafB, is likely involved in catalysis due to its proximity to the sugar-binding site.

Conserved interaction regions and substrate specificity-related residues were identified (Figure 3 and Figure 4). The glycine-rich region (gray box in Figure 3) that interacts with nucleotide phosphates showed substitutions at positions K15 and R20, potentially increasing the positive charge of the N-terminal end of the α2 helix (Figure 4A). Furthermore, the main chain atoms of the I250 and V251 residues at the N-terminal end of the α10 helix appeared to form hydrogen bonds with the beta phosphate (PB). The β9-α9 loop, α9 helix, and α10 helix residues were found to interact with uridine through hydrogen bonds and hydrophobic interactions (Figure 4B). Among these, E254 on the α10 helix is a highly conserved residue in glycosyltransferases that binds UDP (Figure 3, violet box with star) [32]. The galactose moiety of UDP-Gal appeared to form hydrogen bonds with the side chains of R173 and K174 at the N-terminal end of the α7′ helix (Figure 4C). The R/K pair is thought to stabilize the negative charge of the phosphate groups after the sugar moiety leaves UDP-Gal, functioning similarly to the divalent cations present in GT-A glycosyltransferases [23].

Furthermore, residues in the β10-α10 loop were found to form hydrogen bonds with the galactose moiety of UDP-Gal (Figure 4C). The residue E246, which is highly conserved in glycosyltransferases (Figure 3, yellow box with stars), is important for catalytic activity and specificity for monosaccharide sugars. Mutations in this residue are related to the total loss of enzyme activity [23,32]. Additionally, residues from the SD domain contribute to enzyme activity, with H75 and Y52 being important for interaction and substrate specificity [29,30,33,34]. However, D80, a characteristic residue in *Ef*LafB, is likely involved in catalytic activity because of its proximity to the sugar-binding site of UDP-Gal, similar to that reported for WaaG glycosyltransferase from *E. coli* [23]. Taken together, the sequence analysis confirmed that LafB from *E. faecium* retains the key amino acids characteristic of catalytically active glycosyltransferases.

### 2.3. The W193R Mutant and Its Implication in the Structural Integrity of EfLafB

**Figure 5:**
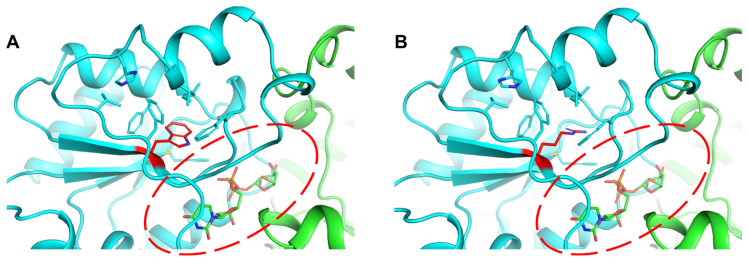
Impact of the W193R mutation on the hydrophobic core and substrate binding of the ND domain in *Ef*LafB. (**A**) Tryptophan 193 (red) in wild-type *Ef*LafB, located near the substrate-binding site, stabilizes the hydrophobic core of the ND domain. (**B**) The substitution of W193 with arginine introduces a positive charge in the hydrophobic core that could destabilize the ND domain and affect substrate binding.

The *E. faecium* HBSJRP18 strain, hypersusceptible to daptomycin, has a mutation at position 193, in which tryptophan (W) is replaced by arginine [5]. This substitution occurs in the hydrophobic core of the ND domain near the substrate-binding site. In wild-type *Ef*LafB, as observed in Figure 5A, W193 (located on the β8 strand) stabilizes the hydrophobic core of the ND domain. AlphaFold3 prediction for *Ef*LafB2.1 revealed a structure nearly identical to that of the wild-type protein, with an RMSD of 0.3 Å for 349 aligned residues. However, the local confidence score of the AF3 model was slightly lower for the region spanning the residues 165–180, which encompasses the mutated residue’s environment (Figure S4). Consistent with this observation, Rosetta FastRelax energy minimization, followed by residue-level interaction energy calculations using Rosetta’s residue_energy_breakdown tool as implemented in Rosetta 371 (2024.09 release), showed that the interaction energies between residue 193 and its surrounding residues were consistently more favorable for the wild-type W193 compared to the mutant R193 in *Ef*LafB2.1 (Figure S4). Together, these observations suggest that the substitution of tryptophan with arginine (Figure 5B) introduces a positive charge, potentially destabilizing the ND domain and, consequently, substrate binding and enzyme activity in synthesizing membrane glycolipids.

### 2.4. The Hypersusceptible Strain Grows More Slowly than the Wild-Type Strain

**Figure 6:**
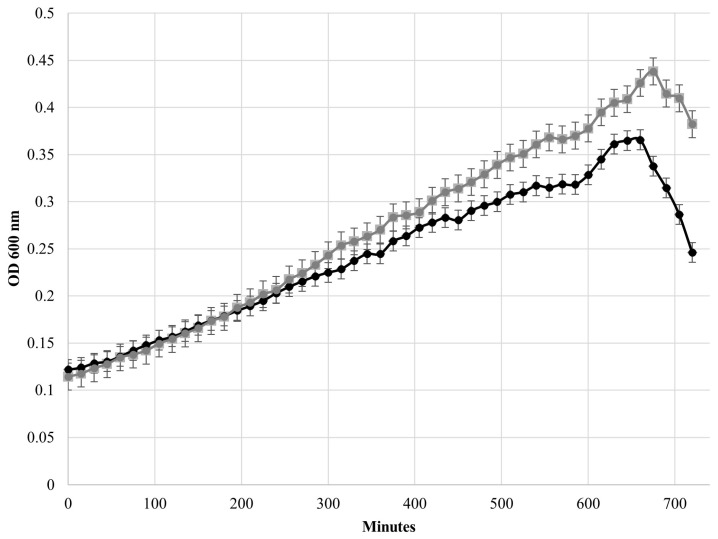
Comparison of the growth curves of the *E. faecium* HBSJRP18_2.7 (gray) and HBSJRP18 (black) strains.

After plotting the growth curves of HBSJRP18 and HBSJRP18_2.7 (Figure 6), we calculated the strains’ doubling times in the conditions tested, which were 360 ± 30 min for HBSJRP18 and 281 ± 11 min for HBSJRP18_2.7, being statistically different (*p* = 0.03).

### 2.5. The Mutated Strain Forms Less Biofilm In Vitro

**Figure 7:**
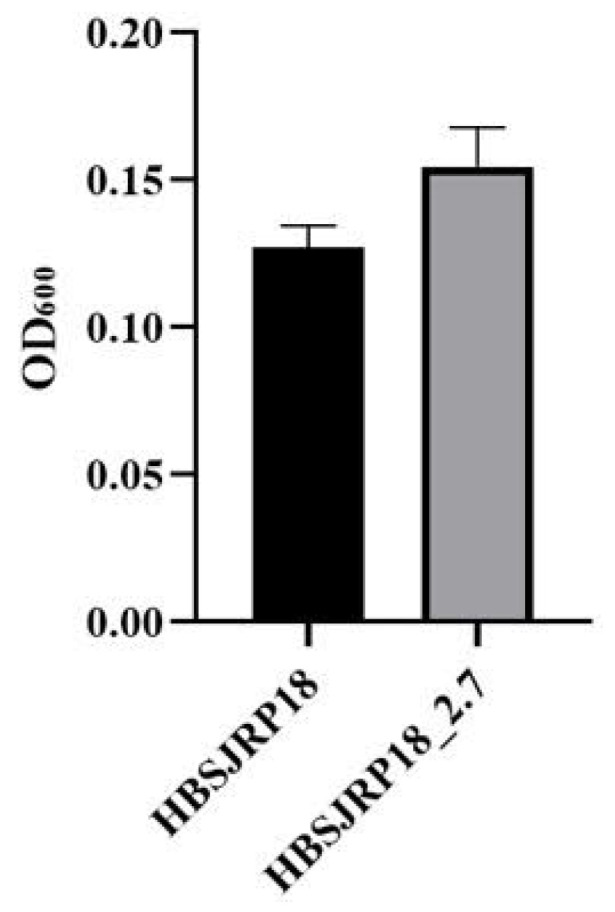
Biofilm formation by *E. faecium* strains HBSJRP18 and HBSJRP18_ 2.7. Results of twelve replicates of each strain (*p* = 0.08).

The hypersusceptible strain formed less biofilm than HBSJRP18_2.7. However, the difference in biofilm biomass between the two strains was not statistically significant (*p* = 0.08) (Figure 7).

### 2.6. Mutation in LafB Impairs Virulence in In Vivo in the Galleria mellonella Model

**Figure 8:**
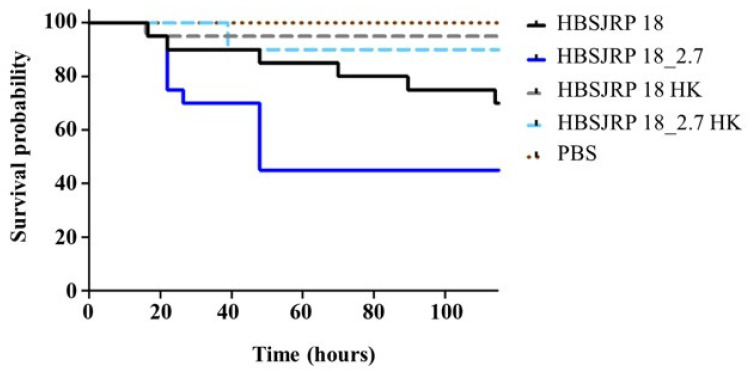
Kaplan–Meier plots of larvae infected with *E. faecium* HBSJRP18 (*lafB* mutation) and HBSJRP18_2.7 (*lafB* restored). The plots shown combine data from three independent experiments. HBSJRP18_2.7 exhibited significantly greater virulence than HBSJRP18, as determined by the Mantel–Cox log-rank test (*p* = 0.0439). HK: heat-killed.

To assess the impact of LafB disruption on infection in an animal model, the *E. faecium* HBSJRP18_2.7 and HBSJRP18 strains were tested in a well-established *Galleria mellonella* invertebrate model. Over the course of several days, we observed that the daptomycin-hypersusceptible strain HBSJRP18 was significantly less virulent in this model than the HBSJRP18_2.7 lineage (Figure 8). The loss of viability of *G. mellonella* was due to active infection, as the control larvae infected with heat-inactivated bacteria showed robust survival.

## 3. Discussion

*E. faecium* contains type I lipoteichoic acid (LTA), which is common among bacteria in the *Bacillota phylum*, such as *Bacillus subtilis*, *S. aureus*, and *Listeria monocytogenes*. Type I LTA is characterized by an unbranched glycerophosphate (GroP) backbone that is generally linked to the bacterial membrane through a glycolipid anchor [35].

In *L. monocytogenes*, LTA synthesis begins in the cytoplasm with the addition of the first sugar (glucose) to diacylglycerol (DAG) in the membrane in a reaction catalyzed by the glycosyltransferase LafA. The second sugar (galactose) is then added to the glycosyltransferase LafB using UDP-galactose (UDP-Gal) as a substrate. Once the LTA anchor (Gal-Glc-DAG) is formed, the enzyme LtaP adds the first GroP molecule to the anchor glycolipid, using phosphatidylglycerol (PG) as the substrate. Finally, the enzyme LtaS elongates the GroP chain, forming the LTA backbone [19].

Previously, our group found a W193R mutation in LafB of the *E. faecium* HBSJRP18 strain, which increases its susceptibility to daptomycin. Trans-complementation with a cloned wild-type gene provided evidence that this W193R mutation is a loss-of-function mutation [5], resulting in either reduced or aberrant LTA biosynthesis. In this study, we characterized biophysically *Ef*LafB and the W193R mutant, used an artificial intelligence tool to predict LafB structure, and investigated the role of the mutation in virulence.

Both wild-type *Ef*LafB and the W193R mutant (*Ef*LafB2.1) were expressed in *E. coli* and purified. The CD spectra showed that both proteins adopt a similar structure, and this observation was further corroborated by the AlphaFold3 models, which showed a similar overall structure for the wild-type and mutant enzymes. However, a stability analysis by thermal denaturation showed a significant decrease in the melting temperature for the mutant enzyme, compared to the wild-type, with a ΔT_M_ of −10 °C. Correspondingly, SEC-MALS analysis identified several species with low molecular mass in the mutant sample. While SDS-PAGE analysis did not reveal evidence of protein degradation, the SEC-MALS findings suggest that these low-molecular-mass species are consistent with the hypothesis that the W193R mutation compromises protein stability and structural integrity. The apparent concentration-dependent nature of this trade-off between structural conservation and reduced stability in *Ef*LafB2.1 was supported by the CD analysis, which indicated that at lower concentrations, *Ef*LafB2.1 appeared to maintain its secondary structure. This instability may have broader implications for bacterial physiology. Given that the *Ef*LafB protein is involved in the biosynthesis of LTA, the reduced stability of the mutant protein could affect proper LTA production, potentially weakening the bacterial cell wall and contributing to the increased susceptibility of *E. faecium* HBSJRP18 to daptomycin [5]. Further studies are necessary to elucidate the exact molecular mechanisms linking *Ef*LafB stability with antibiotic susceptibility.

It is known that evolutionarily, monomers and higher order homo-oligomers of glycosyltransferases are equally ancient. Some studies suggest that the homo-oligomerization of glycosyltransferases offers advantages such as enhanced regulation through transitions between active and inactive protein structures, which depend on the enzyme oligomeric state [36]. Additionally, evidence from some glycosyltransferase families indicates that oligomerization may be necessary for protein function and stability [37,38]. Although our results showed that *Ef*LafB is present as a monomer in vitro, the aggregation tendency of *Ef*LafB2.1 suggests that the mutation disrupts the proper folding without leading to a defined oligomeric state. Further studies are needed to determine whether *Ef*LafB2.1 can adopt an oligomeric form in vivo to compensate for its reduced stability.

From a structural perspective, AF3 suggested that both proteins can fold into a similar tertiary structure. However, the *local* pLDDT score was slightly reduced for *Ef*LafB2.1. Similarly, using a physics-based approach, Rosetta energies computed for this mutant suggested a reduced local stability compared to that of the wild-type protein. These findings are consistent with experimental observations that previously indicated a loss of function [5], with a decrease in growth and virulence and variations in membrane glycolipids, properties that are important for bacterial interactions with the environment and for the activation of the host immune system [39,40]. Additionally, some Gram-positive bacteria, such as enterococci, utilize membrane lipids, mainly LTA, as a survival mechanism against neutrophil-mediated action [39].

Regarding the role of the *lafB* gene mutation in the isolate’s virulence, we demonstrated here that the mutated strain *E. faecium* HBSJRP18 has a higher doubling time than the wild-type HBSJRP18_2.7 and did not reach the same OD as the wild-type strain. Interestingly, Theilacker et al. [18] observed that deletion of the *E. faecalis lafB* homolog *bgsA* did not affect bacterial growth. Nevertheless, considering that the LafB glycoprotein initiates bacterial cell wall LTA formation [18,19], we attribute the observed growth disparity between our strains HBSJRP18 and HBSJRP18_2.7 to the single mutation (C557T/R193W) present in the hypersusceptible strain. This mutation significantly reduced bacterial growth, likely because of its impact on LafB function, which is under investigation by our group.

In contrast to the markedly reduced biofilm-forming ability of a deletion mutant of the *E. faecalis* homologous glycosyltransferase BgsA [17,18], the hypersusceptible *lafB*-defective *E. faecium* strain showed a slight reduction in biofilm formation, which was not statistically significant. D-alanination of LTA increases its positive charge, promoting biofilm formation and bacterial adherence via electrostatic interaction with the negatively charged surface [20,21]. It is possible that the presence of a single mutation, resulting in a non-conservative amino acid substitution, allows for low-level residual function, which may account for some of these differences, or causes a delay in LTA formation, decreasing its content.

The daptomycin-hypersusceptible strain HBSJRP18 was significantly less virulent in the *G. mellonella* model. This is consistent with observations in a mouse bacteremia model, where Theilacker et al. [17] described a significant decrease in the virulence of an *E. faecalis* strain that did not produce BgsA, a protein homologous to *E. faecium* LafB. Theilacker et al. [17] suggested that this loss of virulence could be a consequence of the depletion of DGlcDAG (a molecule synthesized by the BgsA and LafB proteins) or of alterations in the length of LTA, which occur in the cell wall in the absence of the glycosyltransferase BgsA. In our previous study, we demonstrated by FTIR analysis that the mutated strain showed some differences in LTA content. Our group is still working to understand this difference caused by the mutated gene, but the fact is that, based on our current results, the lack of LTA or modifications in its anchor biosynthesis due to defective LafB lead to a less virulent strain in addition to DAP hypersusceptibility, reinforcing the importance of LafB as a pharmacological target.

## 4. Materials and Methods

### 4.1. Cloning and Expression of E. faecium lafB Genes and Protein Purification

The *lafB* coding sequences from *E. faecium* HBSJRP18 and *E. faecium* HBSJRP18_2.7 (GenBank code: QFWO01000106.1, locus_tag: DJ554_13950) were amplified using the following oligos for *lafB* HBSJRP18 (called *Ef*LafB2.1) and *lafB* HBSJRP18_2.7 (called *Ef*LafB, wild-type gene): forward 5′-CAGGGCGCCATGAAGGTATTATTATATTTTGAAAGTGAAAAG-3′, and reverse 5′-GACCCGACGCGGTTACTAGTCCTTGACCTGATTTAC-3′. The amplified sequences were subsequently cloned into the pETM11/LIC expression vector by the ligase-independent cloning (LIC) method [41]. The pETM11*laf*B2.1 and pETM11*laf*B constructs were then introduced into *E. coli* Rossetta (DE3) by heat shock, and cells harboring the cloned genes were grown at 37 °C in lysogeny broth (LB) supplemented with kanamycin (50 µg·mL^−1^) and chloramphenicol (34 µg·mL^−1^) until the cultures reached an optical density at 600 nm (OD_600nm_) of 0.5–1.0. Recombinant protein expression was induced by adding 0.4 mM of isopropyl β-D-1-thiogalactopyranoside (IPTG) (Sigma-Aldrich, St. Louis, MO, USA), followed by cooling the culture to 18 °C. After 18 h, the culture was centrifuged at 4000× *g* for 40 min at 4 °C, and the cells were resuspended in a lysis buffer (50 mM Tris-HCl pH 8.3, 500 mM NaCl, 5% glycerol, 5 mM 2-mercaptoethanol, 0.25% Triton X-100, 0.1 mM PMSF (Sigma-Aldrich, St. Louis, MO, USA)). Ultrasonication was employed to lyse the cells, and the soluble fraction was isolated by centrifugation at 12,000× *g* for 45 min at 4 °C. The soluble fraction was then loaded onto a column pre-equilibrated with lysis buffer (without 2-mercaptoethanol, PMSF, or Triton X-100) containing 3 mL of Ni-NTA Agarose (Qiagen^TM^, Hilden, North Rhine-Westphalia, Germany). Subsequently, proteins were eluted using an imidazole gradient (5, 20, 250, and 500 mM). A second purification step by size-exclusion chromatography (SEC) was performed using a Superdex 200 XK16/600 column (Cytiva, Marlborough, MA, USA) pre-equilibrated with SEC buffer (50 mM Tris-HCl pH 8.3, 150 mM NaCl, 5% glycerol, 5 mM 2-mercaptoethanol) coupled to an AKTA pure system (Cytiva, Marlborough, MA, USA). The purity of the eluted proteins from both purification steps was evaluated by 12% SDS-PAGE. The desired protein concentration was achieved by centrifugation at 1500× *g* using an Amicon Ultra centrifugal filter device with a 30 kDa molecular weight cut-off, and the absorbance at 280 nm was measured using an extinction coefficient of 42,415 M^−1^·cm^−1^. Finally, the samples were frozen at −80 °C for future use.

### 4.2. Size-Exclusion Chromatography Coupled with Multi-Angle Light Scattering (SEC-MALS)

Size-exclusion chromatography coupled with multi-angle light scattering (SEC-MALS) was used to study the oligomeric states of *Ef*LafB2.1 and *Ef*LafB. For the analysis, a 50 μL sample at a final concentration of 2 mg·mL^−1^ was used. The experimental setup consisted of the three-angle light-scattering detector miniDAWN TREOS^®^ (Wyatt Technology, Santa Barbara, CA, USA) and the Optilab T-rEX differential refractometer (Wyatt Technology, Santa Barbara, CA, USA), both coupled to an HPLC system (Waters, Milford, MA, USA) consisting of a pump and a controller (Waters 600, Milford, MA, USA). Protein populations were separated using a Superdex 200 Increase 10/300 GL column (Cytiva, Marlborough, MA, USA) pre-equilibrated with 50 mM Tris-HCl and 200 mM NaCl, pH 7.8. Data collection and analysis were performed using the Wyatt ASTRA 7 software (Wyatt Technology Corporation, Santa Barbara, CA, USA).

### 4.3. Circular Dichroism

The *Ef*LafB2.1 and *Ef*LafB folding and thermostability analyses were carried out using a J-815 spectropolarimeter (Jasco) coupled to a Peltier temperature-control system. Samples in 20 mM phosphate buffer pH 8.0 and 100 mM NaCl, at a final concentration of 0.25 mg·mL^−1^, were used for far-UV spectral recordings spanning from 200 nm to 270 nm at 20 °C. The scanning speed was set at 50 nm.min^−1^ with a spectral bandwidth of 0.5 nm, digital integration time of 2 s, and a 1 mm pathlength quartz cell. For the thermal denaturation assays, a temperature gradient ranging from 10 °C to 90 °C was employed, with consecutive data collection at every 2 °C intervals. The circular dichroism (CD) spectra were converted to mean molar ellipticity per residue (MRW—mean residue weight) according to Equations (1) and (2):

θ_MRW,λ_ = (MRW × θ_λ_)/(10 × d × c) (1)

where MRW = M/(N − 1)(2)

M is the molecular mass of the protein in Daltons, and N is the number of amino acids. θ_λ_ represents the observed ellipticity (degrees) at wavelength λ, d corresponds to the optical path length (cm), and c is protein concentration (g·mL^−1^). The denatured protein fraction (ƒ_d_) was determined using the following Equation (3):

ƒ_d_ = (θ_n_ − θ_obs_)/(θ_n_ − θ_d_) (3)

where θ_obs_ is the ellipticity at 222 nm measured at a specific temperature, and θ_d_ and θ_n_ denote the ellipticities of the denatured and native states, respectively.

### 4.4. Bioinformatic Analysis of EfLafB

De novo modelling of the *Ef*LafB2.1 and *Ef*LafB wild-type protein structures was performed using the AlphaFold3 server [42]. The model reliability was determined using the pLDDT score and Verify3D software (https://saves.mbi.ucla.edu/ accessed on 3 February 2025) [43]. Molecular docking with the potential substrate UDP-galactose (UDP-Gal) was performed using AutoGridFR1.0 Software [44]. The AutoSite option was used to identify and cluster high-affinity points within the ligand-binding pockets, and the docking box was positioned accordingly. An affinity map was generated with default parameters, and a randomized dock was performed with eight independent searches (–nbRuns 8), each using a maximum of 200,000 evaluations of the scoring function (–maxEvals 200,000). Residues directly interacting with UDP-Gal were identified using Discovery Studio Visualizer V21.1.0 (BIOVIA, Dassault Systèmes), and the figures were generated using PyMol v3.1.3.1 (Schrödinger, LLC, New York, NY, USA).

A phylogenetic analysis was performed in MEGA 11.0.13 [45] using prokaryotic sequences of GTB-4 family proteins, selecting one prokaryotic representative from each of the GT1 to GT10 families, as well as LafA (*Listeria monocytogenes*, *Salmonella* sp., and *Enterococcus faecalis*) and LafB from *Enterococcus faecalis*, all sourced from the Carbohydrate-Active-Enzyme (CAZY) database [46].

### 4.5. E. Faecium HBSJRP18 and HBSJRP18_2.7 Growth Curves

To generate the growth curves for the *E. faecium* HBSJRP18 and HBSJRP18_2.7 lineages, each bacterial culture was adjusted in Mueller Hinton cation-adjusted (MHCA) medium to an OD_600nm_ of 0.05–0.1; then, absorbance was measured every 15 min over a period of 12 h using a Spectramax M5 spectrophotometer (Molecular Devices, San Jose, CA, USA). Curves of optical density versus incubation time were constructed for each repetition of the experiment, and data from the logarithmic exponential growth phase of the bacterial cultures were used to calculate the doubling times of each bacterial lineage according to Hall et al. [47]. An ANOVA statistical test was subsequently performed to determine the significance of the differences in the lineages’ doubling times.

### 4.6. Biofilm Formation Ability

Biofilm formation (biofilm mass) by the HBSJRP18 and HBSJRP18_2.7 strains was quantitatively evaluated according to the methodology of Qin et al. [48], with the modifications described by Carrasco et al. [49]. Briefly, an isolated colony from a fresh culture was inoculated into 35 mL of brain heart infusion (BHI) broth supplemented with 0.75% glucose and then incubated at 37 °C for 24 h. After that, the culture was centrifuged at 1500× *g* at 4 °C for 10 min, and the resulting pellet was resuspended in 0.5 mL of BHI broth with 0.75% glucose. Next, 0.05 mL of this suspension was added to 0.45 mL of phosphate-buffered saline (PBS) to reach an optical density of 1.0 at OD_600nm_. The adjusted suspension was then diluted 1:40 in BHI broth with 0.75% glucose, and 0.2 mL of the diluted suspension was added to the wells of a flat-bottomed 96-wells microplate, with at least triplicate wells for each strain. The microplate was incubated at 37 °C for 24 h, followed by removal of the medium from each well and washing for three times with PBS to remove non-adherent cells. The biofilm remaining at the bottom of the wells was allowed to dry at room temperature. For the quantitative evaluation of biofilm mass, a 0.2% crystal violet solution (0.2 mL) was added to each well to stain the biofilms for 15 min. Subsequently, excess and unbound crystal violet were removed by washing three times with PBS, and 0.2 mL of an ethanol/acetone solution (80:20) was added to each well, followed by shaking for 1 min. After homogenization, 0.04 mL of the diluted crystal violet solution was transferred to 0.16 mL of the ethanol/acetone solution, and the absorbance was measured at OD_600_ using a microplate reader (Polaris microplate reader, Celler, Belo Horizonte, Brazil). Statistical analysis of the data was performed using ANOVA.

### 4.7. In Vivo Virulence Assessment

In vivo virulence evaluation was carried out in a *Galleria mellonella* model according to the methodology previously described by Gaca et al. [50]. Briefly, groups of 20 larvae (200–300 mg in weight) were injected with 5 μL of bacterial inoculum containing approximately 2 × 10^6^ CFU. Larvae injected with heat-inactivated *E*. *faecium* (30 min at 100 °C) or PBS were used as negative and vehicle controls, respectively. After infection, the larvae were kept at 37 °C, and survival was recorded at selected intervals for up to 120 h. The experiment was replicated thrice to ensure the robustness and reliability of the results. Kaplan–Meier survival plots were generated, and survival differences were compared using the Mantel–Cox log-rank test.

## 5. Conclusions

In this study, we investigated the structure and function of LafB glycosyltransferase, shedding light on the phenotype of a lineage harboring a W193R mutation in LafB, which renders *E. faecium* hypersusceptible to daptomycin. Through experimental and in silico analyses, we verified that wild-type *Ef*LafB exhibits all the hallmarks of an active glycosyltransferase. It belongs to the GT-B superfamily, specifically related to the GT-4 family, and features two αβ domains that efficiently bind to UDP-Glc. Our computational investigations suggested that the mutation may disrupt the hydrophobic core of the ND domain, potentially impairing substrate ligation. Experimentally, in addition to the previously demonstrated hypersusceptibility to DAP, we demonstrated that this LafB mutation decreases strain virulence in our studied invertebrate model. These findings highlight LafB as a promising target for combating infections caused by *E. faecium*, offering valuable insights into the interplay between LafB structure, its function, and bacterial virulence.

## Data Availability

The data presented in this study are available on request from the corresponding author.

## Supplementary Materials

The following supporting information can be downloaded at: https://www.mdpi.com/article/10.3390/ijms26135935/s1.
