# Supplementary material for: Exploring Daptomycin Hypersensitivity in Enterococcus faecium: The Impact of LafB Mutation on Bacterial Virulence
Source: Int J Mol Sci. 2025 Jun 20;26(13):5935. doi: 10.3390/ijms26135935 (PMC12249671; doi:10.3390/ijms26135935)
Supplement: Supplementary file 1 [file ijms-26-05935-s001.zip › ijms-3667830-supplementary.pdf]

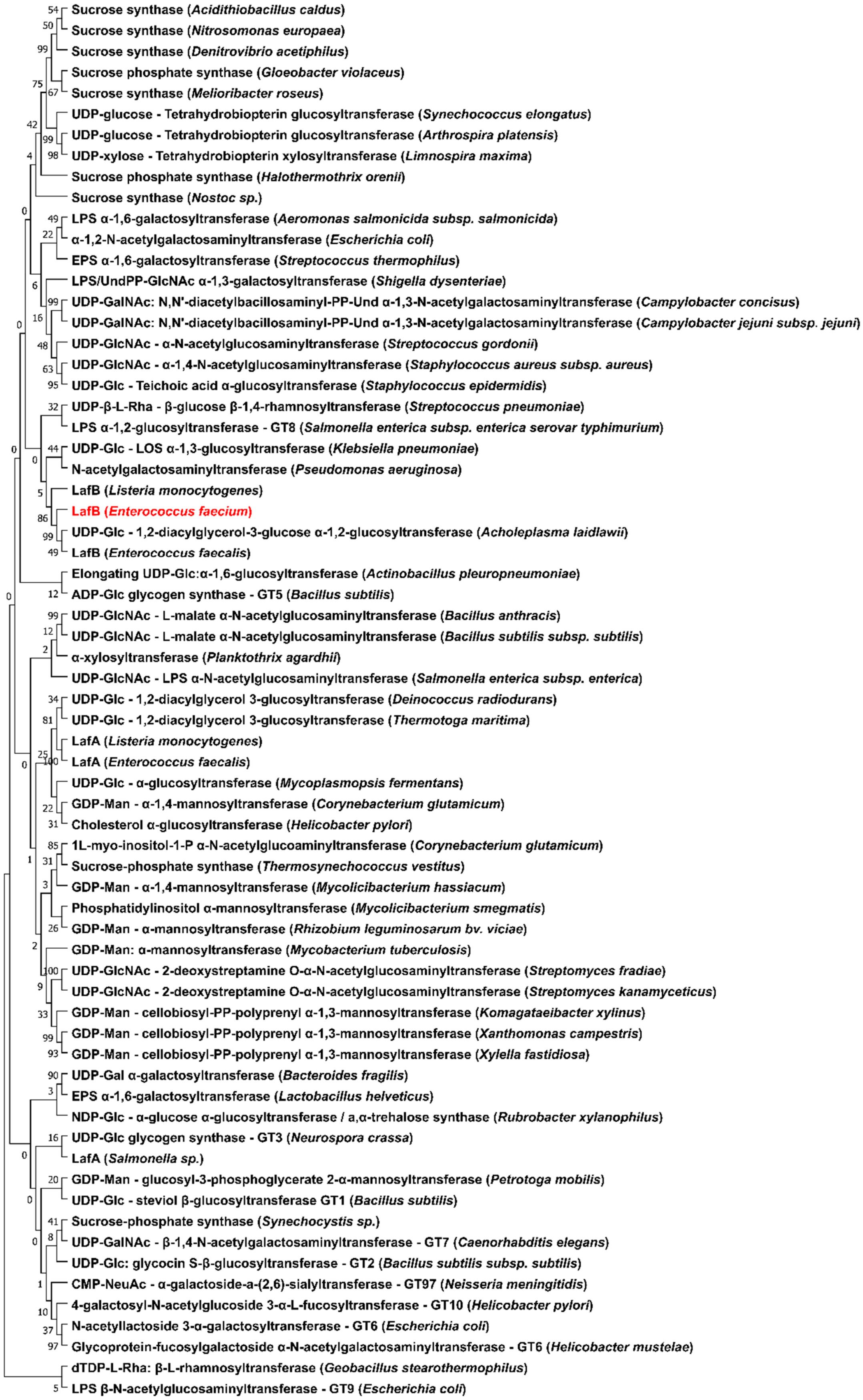

Sucrose synthase (Acidithiobacillus caldus)
Sucrose synthase (Nitrosomonas europaea)
Sucrose synthase (Denitrovibrio acetiphilus)
Sucrose phosphate synthase (Gloeobacter violaceus)
Sucrose synthase (Melioribacter roseus)
UDP-glucose - Tetrahydrobiopterin glucosyltransferase (Synechococcus elongatus)
UDP-glucose - Tetrahydrobiopterin glucosyltransferase (Arthrospira platensis)
UDP-xylose - Tetrahydrobiopterin xylosyltransferase (Limnospira maxima)
Sucrose phosphate synthase (Halothermothrix orenii)
Sucrose synthase (Nostoc sp.)
LPS α-1,6-galactosyltransferase (Aeromonas salmonicida subsp. salmonicida)
α-1,2-N-acetylgalactosaminyltransferase (Escherichia coli)
EPS α-1,6-galactosyltransferase (Streptococcus thermophilus)
LPS/UndPP-GlcNAc α-1,3-galactosyltransferase (Shigella dysenteriae)
UDP-GalNAc: N,N'-diacetylbacillosaminyl-PP-Und α-1,3-N-acetylgalactosaminyltransferase (Campylobacter concisus)
UDP-GalNAc: N,N'-diacetylbacillosaminyl-PP-Und α-1,3-N-acetylgalactosaminyltransferase (Campylobacter jejuni subsp. jejuni)
UDP-GlcNAc - α-N-acetylglucosaminyltransferase (Streptococcus gordonii)
UDP-GlcNAc - α-1,4-N-acetylglucosaminyltransferase (Staphylococcus aureus subsp. aureus)
UDP-Glc - Teichoic acid α-glucosyltransferase (Staphylococcus epidermidis)
UDP-β-L-Rha - β-glucose β-1,4-rhamnosyltransferase (Streptococcus pneumoniae)
LPS α-1,2-glucosyltransferase - GT8 (Salmonella enterica subsp. enterica serovar typhimurium)
UDP-Glc - LOS α-1,3-glucosyltransferase (Klebsiella pneumoniae)
N-acetylgalactosaminyltransferase (Pseudomonas aeruginosa)
LafB (Listeria monocytogenes)
LafB (Enterococcus faecium)
UDP-Glc - 1,2-diacylglycerol-3-glucose α-1,2-glucosyltransferase (Acholeplasma laidlawii)
LafB (Enterococcus faecalis)
Elongating UDP-Glc:α-1,6-glucosyltransferase (Actinobacillus pleuropneumoniae)
ADP-Glc glycogen synthase - GT5 (Bacillus subtilis)
UDP-GlcNAc - L-malate α-N-acetylglucosaminyltransferase (Bacillus anthracis)
UDP-GlcNAc - L-malate α-N-acetylglucosaminyltransferase (Bacillus subtilis subsp. subtilis)
α-xylosyltransferase (Planktothrix agardhii)
UDP-GlcNAc - LPS α-N-acetylglucosaminyltransferase (Salmonella enterica subsp. enterica)
UDP-Glc - 1,2-diacylglycerol 3-glucosyltransferase (Deinococcus radiodurans)
UDP-Glc - 1,2-diacylglycerol 3-glucosyltransferase (Thermotoga maritima)
LafA (Listeria monocytogenes)
LafA (Enterococcus faecalis)
UDP-Glc - α-glucosyltransferase (Mycoplasmopsis fermentans)
GDP-Man - α-1,4-mannosyltransferase (Corynebacterium glutamicum)
Cholesterol α-glucosyltransferase (Helicobacter pylori)
1L-myo-inositol-1-P α-N-acetylglucoaminyltransferase (Corynebacterium glutamicum)
Sucrose-phosphate synthase (Thermosynechococcus vestitus)
GDP-Man - α-1,4-mannosyltransferase (Mycolicibacterium hassiacum)
Phosphatidylinositol α-mannosyltransferase (Mycolicibacterium smegmatis)
GDP-Man - α-mannosyltransferase (Rhizobium leguminosarum bv. viciae)
GDP-Man: α-mannosyltransferase (Mycobacterium tuberculosis)
UDP-GlcNAc - 2-deoxystreptamine O-α-N-acetylglucosaminyltransferase (Streptomyces fradiae)
UDP-GlcNAc - 2-deoxystreptamine O-α-N-acetylglucosaminyltransferase (Streptomyces kanamyceticus)
GDP-Man - cellobiosyl-PP-polyprenyl α-1,3-mannosyltransferase (Komagataeibacter xylinus)
GDP-Man - cellobiosyl-PP-polyprenyl α-1,3-mannosyltransferase (Xanthomonas campestris)
GDP-Man - cellobiosyl-PP-polyprenyl α-1,3-mannosyltransferase (Xylella fastidiosa)
UDP-Gal α-galactosyltransferase (Bacteroides fragilis)
EPS α-1,6-galactosyltransferase (Lactobacillus helveticus)
NDP-Glc - α-glucose α-glucosyltransferase / a,α-trehalose synthase (Rubrobacter xylanophilus)
UDP-Glc glycogen synthase - GT3 (Neurospora crassa)
LafA (Salmonella sp.)
GDP-Man - glucosyl-3-phosphoglycerate 2-α-mannosyltransferase (Petrotoga mobilis)
UDP-Glc - steviol β-glucosyltransferase GT1 (Bacillus subtilis)
Sucrose-phosphate synthase (Synechocystis sp.)
UDP-GalNAc - β-1,4-N-acetylgalactosaminyltransferase - GT7 (Caenorhabditis elegans)
UDP-Glc: glycocin S-β-glucosyltransferase - GT2 (Bacillus subtilis subsp. subtilis)
CMP-NeuAc - α-galactoside-a-(2,6)-sialyltransferase - GT97 (Neisseria meningitidis)
4-galactosyl-N-acetylglucoside 3-α-L-fucosyltransferase - GT10 (Helicobacter pylori)
N-acetyllactoside 3-α-galactosyltransferase - GT6 (Escherichia coli)
Glycoprotein-fucosylgalactoside α-N-acetylgalactosaminyltransferase - GT6 (Helicobacter mustelae)
dTDP-L-Rha: β-L-rhamnosyltransferase (Geobacillus stearothermophilus)
LPS β-N-acetylglucosaminyltransferase - GT9 (Escherichia coli)

Figure S1. Phylogenetic analysis of *Ef*LafB and related glycosyltransferases. Maximum-likelihood phylogenetic tree of glycosyltransferases (GTs) from different bacterial species, including *Enterococcus faecium Ef*LafB (highlighted in red). The tree was constructed using representative sequences from GT-4 family enzymes, with bootstrap values shown at branch nodes. The clustering of *Ef*LafB with LafB from *Listeria monocytogenes* and *Enterococcus faecalis* suggests a conserved evolutionary relationship among these glycosyltransferases. The analysis provides insights into the functional classification of *Ef*LafB within the GT-B superfamily, specifically within the subgroup of UDP-sugar-dependent glycosyltransferases involved in glycolipid biosynthesis.

>WP_002287604.1 LafB (*Enterococcus faecium*)

MLYYISEGGRKVKVLLYFESEKMLAKSGIGRALDHQKRALTEVGISYTLDEKEDYDILHI
NTYGINSHNMVNKARREGKKVVYHAHSTEEDFRNSFIGSNQLSPIVKKYLVGLYQKAD
YLITPTPYSKQLLESYGIRVPIQAISNGIDLEKYHPDPMKEQKFREYFKLSPDQKVIICVGL
FFERKGIIDFVEIAKKMPEYTFIWFGHVPMYSIPRNIRKIVKEDHPENVLFPGYIRGEIIEG
AYSGADLFFFPSYEETEGIVVLEALASKQNVLVRDIPVYNGWLEDSKNCYMGKTNEEFI
RLIQQITNQELPSTTQAGYQTAKERSINKIGEELKNVYESVLNEKVSSKLKKVNQVKD

>AAL83700.1 UDP-Glc - 1,2-diacylglycerol-3-glucose α-1,2-glucosyltransferase (*Acholeplasma laidlawii*)

MKVLLYSQKQSMLKKSGIGRAFYHQKRALEAVGIEYTTDPKDTYDLVHVNIAHSNKIK
KFRKKYPVIVHGHSTVQDFRRSFAFWRVIAPFFYKHLQNIYGIADLIITPTRYSKFLIESM
HVVKSPVVALSNGIDLDAYEYKQENVDAFRKHFDLEPNQKVVIGVGLLFERKGIHDFIE
VARTMPNVTFIWFGNLSKLATTHFIRKRIKNKPKNMIMPGYVDGAVIKGAFSGADCVFF
PSYEETEGIVVLEGLASKTPVVLRDIPVYYDWLFHKEHVLKGHNNFEFSKLIEKVLHED
QTEMIENGYKIVQDRSIEKIGEGLKQAYQEVIKIKR

>AIA55343.1 Sucrose synthase (*Acidithiobacillus caldus*)

MIEALRQQLLDDPRSWYAFLRHLVASQRDSWLYTDLQRACADFREQLPEGYAEGIGPLE
DFVAHTQEVIFRDPWMVFAWRPRPGRWIYVRIHREQLALEELSTDAYLQAKEGIVGLGA
EGEAVLTVDFRDFRPVSRRLRDESTIGDGLTHLNRRLAGRIFSDLAAGRSQILEFLSLHRL
DGQNLMLSNGNTDFDSLRQTVQYLGTLPRETPWAEIREDMRRRGFAPGWGNTAGRVR
ETMRLLMDLLDSPSPAALESFLDRIPMISRILIVSIHGWFAQDKVLGRPDTGGQVVYILD
QARALEREMRNRLRQQGVDVEPRILIATRLIPESDGTTCDQRLEPVVGAENVQILRVPFR
YPDGRIHPHWISRFKIWPWLERYAQDLEREVLAELGSRPDLIIGNYSDGNLVATLLSERL
GVTQCNIAHALEKSKYLYSDLHWRDHEQDHHFACQFTADLIAMNAADIIVTSTYQEIAG
NDREIGQYEGHQDYTLPGLYRVENGIDVFDSKFNIVSPGADPRFYFSYARTEERPSFLEPE
IESLLFGREPGADRRGVLEDRQKPLLLSMARMDRIKNLSGLAELYGRSSRLRGLANLVII
GGHVDVGNSRDAEEREEIRRMHEIMDHYQLDGQLRWVGALLDKTVAGELYRVVADGR
GVFVQPALFEAFGLTVIEAMSSGLPVFATRFGGPLEIIEDGVSGFHIDPNDHEATAERLAD
FLEAARERPKYWLEISDAALARVAERYTWERYAERLMTIARIFGFWRFVLDRESQVMER
YLQMFRHLQWRPLAHAVPME

>ACE62348.1 Elongating UDP-Glc:α-1,6-glucosyltransferase (*Actinobacillus pleuropneumoniae*)

MENNIDLNVYFCFVNRPCTGGDFVNLDHVRTLRKLGINASILLAGNQSEEIVNSFGSLPV
VILNEEIEFSSQDIFIVPEVMQVLYDLASKMTVFPRMIMHNQNPFYTGYGFLSAQHINEH
RLERIIVPSSYTKYKLQEIGVTKPIDIIHPYIPDYFKPAEKQREVIQIAFSRRKRSAEFDIFKF
YFLSLYSHKHSVNFVNIQGLTREEVAKVMSEAAIFISFAERESLGLMTLEAMASGCHVIG
FSGYTDIYNNEVIDDSVGDWIGEGEYTLFAQKVCQAIDDFVNGKMNPKIENGLRLIEQR
FRIRHFEQEVKRVYGNIFDYDLENSRS

>ABO88293.1 LPS α-1,6-galactosyltransferase (*Aeromonas salmonicida subsp. salmonicida*)

MKKKVLFLMETLGGGGAEKVLVNIVNSLDDIKYDVTLLLLKREGMYLSKIPPHVKIKY
LLDAEPTGLVRRGMMYSLKKRWLNAVLSKPWLANMSFPEEYDVGVSFLEGDSSLLLSH
LDGPKKKIAWVHIDLEKHHTLPREIEKVAYESMDQIICVSEGSKHSVLSLYPALNDITSVI
YNPIDLKKIIDGGEQKIRVGSGLNVLAVGRLLNDQKAFDVLLDAHRINISAGLNYHLTIL
GEGSDRSTLEAYITEHQLGDNTSLLGFKDNPYPYIAGCDIFVISSHYEGYPVVLVEAMTL
GKPIVSTDCTGPKEALSNGEFGHLVPIANAQALADGLKMLLENDAIRERYAELSKQRSE
FFSFERSMRDIETLLNT

>ADE62102.1 UDP-glucose - Tetrahydrobiopterin glucosyltransferase (*Arthrospira platensis*)

MTPTSWKLLFISTPVGPLGSGLGGGVELTLLNMAKALKSRGHDITVVAPSGSVLESLSIIE
IPGELQPIAQNQDRDSLITIPENSVLGNMWEYGRQVQTNYHAIVNFAFDWLPFYLTPFFD
TPIAHCVSMASLISALDQIVGQVMKQFPGTVGFHSHTQAATFGSDLDYACLGSGLEMER
YQFCEQPHQQLAWMGRISPEKGLEDAIAAADKTGIPLEIFGKIQDDQYWQNILNTYPNA
PLNYRGFLNTDELQQGLRQCRGLLMTHRWVEAFGNVAIEALACGVPVISYRRGGPAEI
VRDGETGWLVEPDSVTGLVDAIAKFEQIDRRQCRAVAEKEYSLAALGDRLEKWLSDVT
RLKN

>AAP25494.1 UDP-GlcNAc - L-malate α-N-acetylglucosaminyltransferase (*Bacillus anthracis*)

MKLKIGITCYPSVGGSGVVGTELGKQLAERGHEIHFITSGLPFRLNKVYPNIYFHEVTVN
QYSVFQYPPYDLALASKMAEVAQRENLDILHVHYAIPHAICAYLAKQMIGERIKIVTTLH
GTDITVLGSDPSLNNLIRFGIEQSDVVTAVSHSLINETHELVKPNKDIQTVYNFIDERVYF
KRDMTQLKKEYGISESEKILIHISNFRKVKRVQDVVQAFAKIVTEVDAKLLLVGDGPEFC
TILQLVKNLHIEDRVLFLGKQDNVAELLAMSDLMLLLSEKESFGLVLLEAMACGVPCIG
TRVGGIPEVIQHGDTGYLCEVGDTTGVADQAIQLLKDEELHRNMGERARESVYEQFRSE
KIVSQYETIYYDVLRDDKNGKI

>AIY93568.1 UDP-GlcNAc - L-malate α-N-acetylglucosaminyltransferase (*Bacillus subtilis subsp. subtilis*)

MRKLKIGITCYPSVGGSGIIATELGKQLAEKGHEIHFITSSIPFRLNTYHPNIHFHEVEVNQ
YAVFKYPPYDLTLASKIAEVAERENLDIIHAHYALPHAVCAYLAKQMLKRNIGIVTTLHG
TDITVLGYDPSLKDLIRFAIESSDRVTAVSSALAAETYDLIKPEKKIETIYNFIDERVYLKK
NTAAIKEKHGILPDEKVVIHVSNFRKVKRVQDVIRVFRNIAGKTKAKLLLVGDGPEKSTA
CELIRKYGLEDQVLMLGNQDRVEDLYSISDLKLLLSEKESFGLVLLEAMACGVPCIGTNI
GGIPEVIKNNVSGFLVDVGDVTAATARAMSILEDEQLSNRFTKAAIEMLENEFSSKKIVS
QYEQIYADLAEPE

>CAH07087.1 UDP-Gal α-galactosyltransferase (*Bacteroides fragilis*)

MRDGKPIEILHIVGTRPVGGIGALLKNINTSIDLNKFHFTYVFSADSNIGDFDNYVRKLG
SDIVVFPSYHLKYLFLYLKVIFCFYKRNAKKYDIIHVHSANTGVLDLLFAKIYGIRIRILHS
HSTKYSSKKIRSIRNYFLQFPTIYLANTYFACGYKAAEFLFGKKRLEKVYIIHNAIFSKKFI
YNATVRERVRVELKIENDCLLLGHIGNFTKEKNHGFLIDILEELLNINDNVKLLLVGDGQ
LRSEIEEKVKLKRLQKYVCFLGRRTDISELLQAMDFLIFPSFFEGLPVTLIEAQASGLRCV
VSDRITLETKITNNISYLNLNRDPSTWAEVVWKLFEDKTFIRKDTSKEICESGYDIHLESE
YLERIYLNLLHK

>ALF48116.1 UDP-GalNAc: N,N'-diacetylbacillosaminyl-PP-Und α-1,3-N-acetylgalactosaminyltransferase (*Campylobacter concisus*)

MARIGFLSHADMSIHFFRRPIMQALKDMGHEVFAIAPKGNFTDELAKSFHAVTYELDKA
SLNPLTVINNSKKLSQILGELNLDLLQTGAHKSNVFGTFAAKNAGIKYVINLVEGLGSFY
IDDDIKTKAVRFVMESLYKISFAKADACVFVNNSDADYMISKNLIDKSKVYRIKSVGVD
TAKFDPAITQAVDLGEKKVILMIARAMWHKGVREFYEAAEILNGYKNCEFVFVGEGFA
GNKSTADESFLKGGKVRYLGARNDIPQLLKASYLLALPSYKEGFPRTVLEAMSMAKAV
VASDVTGCNEAVKDGYNGLLCKVKDANDLAGKIKILLDDEELCARLGANGRSWVVSE
FDEKQIAKRYIEIYRKFIDV

>sp|Q0P9C9| UDP-GalNAc: N,N'-diacetylbacillosaminyl-PP-Und α-1,3-N-acetylgalactosaminyltransferase (*Campylobacter jejuni subsp. jejuni*)

MRIGFLSHAGASIYHFRMPIIKALKDRKDEVFVIVPQDEYTQKLRDLGLKVIVYEFSRAS
LNPFVVLKNFFYLAKVLKNLNLDFIQSAAHKSNTFGILAAKWAKIPYRFALVEGLGSFYI
DQGFKANLVRFVINSLYKLSFKFAHQFIFVNESNAEFMRNLGLKENKICVIKSVGINLKK
FFPIYVESEKKELFWKNLNIDKKPIVLMIARALWHKGVKEFYESATMLKDKANFVLVG
GRDENPSCASLEFLNSGAVHYLGARSDIVELLQNCDIFVLPSYKEGFPVSVLEAKACGK
AIVVSDCEGCVEAISNAYDGLWAKTKNAKDLSEKISLLLEDEKLRLNLAKNAAQDALQ
YDENIIAQRYLKLYDRVIKNV

>CAF19117.1 1L-myo-inositol-1-P α-N-acetylglucoaminyltransferase (*Corynebacterium glutamicum*)

MGMRVAMISMHTSPLQQPGTGDSGGMNVYILSTATELAKQGIEVDIYTRATRPSQGEIV
RVAENLRVINIAAGPYEGLSKEELPTQLAAFTGGMLSFTRREKVTYDLIHSHYWLSGQV
GWLLRDLWRIPLIHTAHTLAAVKNSYRDDSDTPESEARRICEQQLVDNADVLAVNTQEE
MQDLMHHYDADPDRISVVSPGADVELYSPGNDRATERSRRELGIPLHTKVVAFVGRLQ
PFKGPQVLIKAVAALFDRDPDRNLRVIICGGPSGPNATPDTYRHMAEELGVEKRIRFLDP
RPPSELVAVYRAADIVAVPSFNESFGLVAMEAQASGTPVIAARVGGLPIAVAEGETGLLVD
GHSPHAWADALATLLDDDETRIRMGEDAVEHARTFSWAATAAQLSSLYNDAIANENVD
GETHHG

>BAB97862.1 GDP-Man - α-1,4-mannosyltransferase (*Corynebacterium glutamicum*)

MEIIRPMRVAIVAESFLPNVNGVTNSVLRVLEHLKANGHDALVIAPGARDFEEEIGHYLG
FEIVRVPTVRVPLIDSLPIGVPLPSVTSVLREYNPDIIHLASPFVLGGAAAFAARQLRIPAIA
IYQTDVAGFSQRYHLAPLATASWEWIKTVHNMCQRTLAPSSMSIDELRDHGINDIFHWA
RGVDSKRFHPGKRSVALRKSWDPSGAKKIVGFVGRLASEKGVECLAGLSGRSDIQLVIV
GDGPEAKYLQEMMPDAIFTGALGGEELATTYASLDLFVHPGEFETFCQAIQEAQASGVP
TIGPRAGGPIDLINEGVNGLLLDVVDFKETLPAAAEWILDDSRHSEMCAAAWEGVKDK
TWEALCTQLLQHYADVIALSQRIPLTFFGPSAEVAKLPLWVARALGVRTRISIEA

>AAF10795.1 UDP-Glc - 1,2-diacylglycerol 3-glucosyltransferase (*Deinococcus radiodurans*)

MNPLRIGLFTDTFLPDQNGIVTSVCLLSDELRALGHQVDVVAPRFPEHLDRRPDVRRVA
SVRYPLLPTYRLAWPGRRSFEQRYDLIHTHTPLTLGLAGARLARKWGVPHVATYHTHLE
AYTHYVPGMTQLNRAVHFMPKVVGRLYGEADAVITPTAGTLDTLRQAGITDAVVIPTSI
DPAVLEAAPPIASPWPPGTRRLLTVGRLAREKRFDLVLDTLADLPGAHLVILGEGPERTH
LEDHAQRLGVAEHVTFVGVRPWTEIGAYYRLAELFVFASDTETQGLVLQEAQLMGVPV
VAVGARGTLTGVQSGYSGYLTAPGDVPELVRRARELLESPEQYARFSRQAREFGGRTTP
AGVARQVLAVYARTLGLPPESPDDPRADGLSAPAEAAGRPRNNPVYGR

>ADD69694.1 Sucrose synthase (*Denitrovibrio acetiphilus*)

MNLSNKELEGLDEIISDHREDFCPFLGRIEEEDKQFFLSSEMKEMYAGDTVPDFIASLQE
AVKMPGQIYFATRASIGEWAFVTVFTDTLDYMEVSPTEYQEAKEKTVLGENAAWMPSV
DLKPFNRDFPKPSSADFIGKGVEFLNRHQSSRIFMNPEKGLKQLLDFLRVHKYDGRQLM
LNNRIDSVDKLKKALKKAQALLKNKSDETEWEEVESDMAHLGFEPGWGKKLGYVKE
FLALLSDILAAPEPVVLEKFLDRIPMIFSLVVLSPHGFFGQAGVFGKPDTGGQVVYILDQ
VKALEHELKSRLDEKGLDITPKILVVTRLIPEAEGTNCDMEEELIRGTDNCHIVRVPFRD
ESGEVVRQWISRFRIWPYLERFSTEAQNIILSKLQGNPDLIIGNYSDGNLVASLIAQRLGV
TQCTIAHALEKTKYLYSDLYWQDNNDKYHFACQYTADLISMNYSDFIITSTYQEIAGTN
DSVGQYESYMNYTLPGLYRVVNGIDVFDPKFNVVSPGAAPDIFFSYKSKDRFPEHIEEIE
SILFEDNLEGSRGSLADPDKPLIFTMARLDKIKNLTGLVRWFGENEELRKTANLLVIGGF
VDESLSSDDEEREQIRIMHSVIDELGLDGSVRWVGAHLGKRMTGEFYRYVADRKGVFV
QPALFEAFGLTIIEAMSSGLPVFATVYGGPSEIIEDGKSGFTLDPNKGDECAEKLLEFIQKC
QSDPGHWIKISDNALKRVEERYNWPLYAKRLMTFARVYGFWKFVTNLEREETVRYLEM
LYGMVYRRLADPKEY

>APK80348.1 α-1,2-N-acetylgalactosaminyltransferase (*Escherichia coli*)

MKVGFFLLKFPLSSETFVLNQITAFIDMGFEVEIVALQKGDTENTHVAWVKYNLAARTR
WLQDEPAGKVAKLRHRASQTLRGIHRKNTWQALNLKRYGAESRNLILSAICGQVATPFR
ADVFIAHFGPAGVTAAKLRELGVIRGKIATIFHGIDISSREVLNHYTPEYQQLFRRGDLM
LPISDLWAGRLQKMGCPREKIAVSRMGVDMTRFSPRPVKAPATPLEIISVARLTEKKGLH
VAIEACRQLKEQGVAFRYRILGIGPWERRLRTLIEQYQLEDVVEMPGFKPSHEVKSMLD
DADVFLLPSVTGADGDMEGIPVALMEAMAVGIPVVSTLHSGIPELVEADKTGWLVPEN
DARALAQRLAAFSQLDTDELAPVVKRAREKVEHDFNQQVINRELASLLQAL

>AAR99609.1 dTDP-L-Rha: β-L-rhamnosyltransferase (*Geobacillus stearothermophilus*)

MLQKLIQILRRNEYVKNVYKNTVSNFIETSIPEITPFNARTSSIKGKRLNLLVPSINQEHMF
GGISTALKLFEQFDNKKFKKRIILTDATPNPKDLQSFKSFKYVMPEEDKDFALQIVPFNDR
YNRTIPVAKHDIFIATAWWTAYAAQRIVSWQSDTYGIPPNKILYIIQDFEPGFYQWSSQYV
LAESTYKYRGPQIAVFNSELLKQYFNNKGYNFTDEYFFQPKINTTLKNYINDKRQKEKII
LVYGRPSVKRNAFTLIVEALKIFVQKYDRSNEWKIISVGEKHKDIALGKGIHLNSLGKLT
LEDYADLLKRSSIGISLMISPHPSYPPLEMAHFGLRVITNKYENKDLSNWHSNIVSLEQLN
PENIAETLVELCMSFNNRDVDKKESSNMMFYINEFNEFSFIKEIEEKL

>BAC91548.1 Sucrose phosphate synthase (*Gloeobacter violaceus*)

MSDLIQTVIDSEEKADLRQFIGLLYQQEKRYLLRSDILRVFSEYCSQAQKEDSFFSGSLLG
QLIYFTQEFILENESLCLVLRPVIARQEYCRINREDLRVEPLSIQEYLDLCDQTAGRFRPQ
DGDVLELDFQPFYDFSPSIRDSKNIGKGVQFLNRFLSSKLFQDPGRWQQRLFQFLRLHR
HNGSQLLINERIQTLSQLSAQLKRVIAFVSARRPEEPYANFRFDLQSLGFEPGWGNTAAR
VRETLEILDALLDSPDHETLEAFISRIPMVFRVVLVSPHGWFGQEGVLGRPDTGGQVVY
VLDQARSLEKQLIEDHTLAGLEPNPKVVILTRLIPNNDGTRSNQRLEKVYGTDNVWILR
VPLREFNPAVTQNWISRFEIWPYLESFAIDSEKELMAELRGRPDLIVGNYSDGNLVAFLL
ARRLGVTQCIIAHALEKAKYAYSNLQWEELDEQYHFSLQFTADLIAMNAANFVVTSTY
QEIAGTADSVGQYESHRTFTMPDLYHVVSGIDLFNPKFNVVPPGVNENIYFPYTRAEDRT
PGDRERLEQLLFSLDDPDQAYGHLVDPGKRPLFSMARLDRIKNLTGLAECFGRSPALQE
RCNLILVAGKLRAEDSTDREEIAEINRLYEIIDRYGLDGKIRWLGVRLAKVDSGEIYRVIA
DRQGIFVQPALFEAFGLTILESMISGLPTFATRFGGPLEIIQDGVNGFLINPNALEETAEKL
LEFVSKCEANPAYWQQISERAVQRVYSTYTWKIHTTRLLTLARIYGFWNYGSQENREDL
LRYVEMLFYLLFRPRAQKLLERHFQRDSIE

>ACB11221.1 Sucrose phosphate synthase (*Halothermothrix orenii*)

MTRIKHVAFLNPQGNFDPADSYWTEHPDFGGQLVYVKEVSLALAEMGVQVDIITRRIK
DENWPEFSGEIDYYQETNKVRIVRIPFGGDKFLPKEELWPYLHEYVNKIINFYREEGKFP
QVVTTHYGDGGLAGVLLKNIKGLPFTFTGHSLGAQKMEKLNVNTSNFKEMDERFKFH
RRIIAERLTMSYADKIIVSTSQERFGQYSHDLYRGAVNVEDDDKFSVIPPGVNTRVFDGE
YGDKIKAKITKYLERDLGSERMELPAIIASSRLDQKKNHYGLVEAYVQNKELQDKANLV
LTLRGIENPFEDYSRAGQEEKEILGKIIELIDNNDCRGKVSMFPLNSQQELAGCYAYLAS
KGSVFALTSFYEPFGLAPVEAMASGLPAVVTRNGGPAEILDGGKYGVLVDPEDPEDIAR
GLLKAFESEETWSAYQEKGKQRVEERYTWQETARGYLEVIQEIADRKDEEDEGGSLNIP
DYFTNPGASNDEKLLDTFNKLWKE

>AAD07485.1 Cholesterol α-glucosyltransferase (*Helicobacter pylori*)

MVIVLVVDSFKDTSNGTSMTAFRFFEALKKRGHVMRVVAPHVDNLGSEEEGYYNLKER
YIPLVTEISHKQHILFAKPDEKILRKAFKGADMIHTYLPFLLEKTAVKIAREMQVPYIGSF
HLQPEHISYNMKLGWFSWFNMMLFSWFKSSHYRYIHHIHCPSKFIVEELEKYNYGGKK
YAISNGFDPMFRFEHPQKSLFDTTPFKIAMVGRYSNEKNQSVLIKAVALSKYKQDIVLLL
KGKGPDEKKIKLLAQKLGVKAEFGFVNSNELLEILKTCTLYVHAANVESEAIACLEAIS

VGIVPVIANSPLSATRQFALDERSLFEPNNAKDLSAKIDWWLENKLERERMQNEYAKSA
LNYTLENSVIQIEKVYEEAIRDFKNNPHLFKTLS

>AAX20104.1 UDP-Glc - LOS α-1,3-glucosyltransferase (*Klebsiella pneumoniae*)

MSKFRLALVRQKYRPDGGAERFVSRALEALDSSHLQLNVITREWQGPVKPDWQIHICN
PRKWGRISRERSFANAARELWQRESFDLVQSHERIPGCDLYRAGDGVHRRWLQQRSRIL
PAWKSRLLFADRYHRYVMQAEREMYEDSHLRGVICNAEMIKREIIEDFGLPAEKIHVIY
NAIDNQRFLPPDEETFAALRAKWQLPLQATCLIYVGSGFERKGLAAAIRAIAPTDRYLLV
VGKDKDQPRYQALAKSLNCGARVRFFGMQSETLPFYQMADGLLLPTLYDPFPNVILEA
MACGLPVITTTGCGGAEFIVDGHNGYVCDALDIPALQQAVMALPARALSSAEGGHARE
RIMACTSERLSTQLLSLYQDLVN

>AAC44373.1 GDP-Man - cellobiosyl-PP-polyprenyl α-1,3-mannosyltransferase (*Komagataeibacter xylinus*)

MNSKKRGDETLKVLHICRQFSPSVGGLEDSLLNLARSQRQRLGIDAEVLTLDTVFGRPG
KLPHRDVVDGIPVTRLAWRGSTKYPLAPQVLRHIGGFDLLHVHAIDFFFDFLAWTWPL
HRKTMIASTHGGFFHTGALRRIKEIWFRTITPISVRAYKKIVACSYSDADLFRHVAAGRLI
TIENGINQTRFRDAASRTPNRTILAFGRFAVHKRLKLLFQLVALLRAYNSGWNIIVAGQDS
NLTADDLRAQARACGIEDSLRIVSGPSDAELRGLMGEASFFGCLSAHEGFGLAAVEAMS
AGLVPILSNITPFARLMQQGAAGVMVNPDNLAPGAREAEDMAAALPETADALRARNM
DVASRYDWHSVAHEYARLYQQVLGRALPEANMAAAGAE

>CAC07465.1 EPS α-1,6-galactosyltransferase (*Lactobacillus helveticus*)

MKKYRILVWGFTENMGGVENVILNYYQNFDNRIAIDFMAESKKKMAYEDLLRRSGSR
VFHLPRKRYHLLEYYQALKRFFSEHGSDYDCVWSNVNNLSNIDILVYAKRFGVKRRIIH
SHNSKNMFKGIARYQRGIMQWKNKFIISKYATDYWACSKDAADYVFPAKVLSQVKIIRN
AIEVSKYRFDEQKRKKIRKQYNLEGHFVIGNVGRLHFQKNQIYILKILKKIKNDIPTAKL
VFVGDGPDKNNLYDVAEKLNVKDDVIFAGSQAKIEDWYSAFDIFLFPSLFEGFGNALIE
AQANGLMILASQNVIPKETIINSNYKMIPLDKGSNYWAKIIVDNFKVFNHKIPRELDENI
KYNFENKGYNSETNAVKVEDLLLKDKSKHG

>ADE62104.1 UDP-xylose - Tetrahydrobiopterin xylosyltransferase (*Limnospira maxima*)

MPQKSWKLLFISTPVGPLGSGLGGGVELTLLNMAKALHSRGHDITVVAPSGSVLESLSVI
EISGKLQPIAQNQDRDSLITIPENSVLGNMWEYGRQVQTNYHAIVNFAFDWLPFYLTPFF
PTPIAHWVSMGSLISALDQIVVEVMKQFPGTVGFYSHTQAATFGSDLGYACLGSGLEM
DRYQFCDQPHQQLAWMGRISPEKGLEDAIAAADKTGIPLAIFGKIQDEQYWQNILNTYP
NAPLNYRGFLNTDELQQGLRECRGLLMTHRWVEAFGNVAIEALACGVPVISYRRGGPA
EIVRDGETGWLVEPDSVTGLVDAIAKLEQIDRRQCRALAEKEYSLVALGDRLETWLSDV
IALKN

>AFN74551.1 Sucrose synthase (*Melioribacter roseus*)

MIKDIYKTAETFHNDFYDFLKAVSTQPKKLMITGELINLYVASGYDKNSGLYEFIEKIQET
ISLDHSVILDVRIKIASIKFYRISLEEFLIEEISSKEFLIYKETVAKPDTLNTTLNLNFKPFYD
KSPAVRDIKYIGSGVEYLNRFLSSQMFTNEERWKKNLFDFIRLHNFNGEQLILNDRIKDT
KHLNNQINAALAKLGNHPANTPYENIKHILQELGFEKGLGKDAGTITHNLNLLDQLLNS
PDHNALAEFISSIPMILNIAIISPHGFFGQEGVLGLPDTGGQVVYILDQVKALEKQLIDSL
KKSGLNLLPKIIVLTRLIPNARGTTCNQRLEKIYGAKNSWILRVPFREYNKRVTDEWISRF
EIWPYLEDFAEDSYTALLAEFKKRPDLIIGNYSDGNLVAYLLAKKFKVTQCGIAHALEKS
KYLYSALYWYDLEKYYHFSMQFTADLLAINSADFLITSSFQEIAGTEKSIGQYESYMHFT
MPGLYRVENGVNPFHVKFNIVSPGVNEKIYFPYPKTKWRLKETKRRIENLFFSNSEDPD
VIGWLDNPEKTPIFTMSRLDRIKNISFLVRCFGESEELQQTSNLIVVAGKIDETMTDDYEE
KEQIRLMHELITKYKLHNKIRWIGKLLPKDESGEAYRIIAERRGIFVQPALFEGFGLTVLE
AMTSGLPVFATKYGGPLEIIQNGVNGFHIDPVNQEETTEKIVRFLSDSYIDSSVWDKLSK
AAIKRVTEKYSWKLYSKRLLSLAKLYGFWKYATNLEHEDINAYLDLIYHTIYKSRAKILL
EEHMKR

>AAK46073.1 GDP-Man: α-mannosyltransferase (*Mycobacterium tuberculosis*)

MRVVQVANFYGPRSGGLRTAVDRLGAEYCASGHEVFLIVPGARTERHLLRTGVVRITLP
AKHIPYTGGYRAVMPGAVRTVLETLRPDALEVSDRLTLRSLGRWGREHGVTTVMISHE
RLDRFAGQLLPRRAAQKFADFANARTAANYDTVVCTTGFAREEFDRIGATNTVTVPLGV
DLKTFHPRRRCARVRQHWATPTQILLVHCGRLSVEKHADRSIDALAALCDAGVDARLVI
AGEGPLRARLERKATGLPIDFTGFISDRHAVAGLLASADVALAPGPHETFGLAALESLAC
GTPAVVSRTSALTEIITADSGACADNRPEAIAHAVRTIVSRPERHRRRCARRRAEIFTWQR
AAASMLATLGAMAVSTRCGDTQDTA

>VCT92672.1 GDP-Man - α-1,4-mannosyltransferase (*Mycolicibacterium hassiacum*)

MRIALLSYRSKTHCGGQGVYVRHLSRELAELGHDVEVFSGQPYPEGLDPRVRLTKVPSL
DLYREPDPFRIPRPSEIKTSIDLEELLTTWTAGFPEPKTFSLRAARVLAGRRGDFDVVHDN
QCLGTGLLQIAKMGFPLVATVHHPITRDREVEVAAARWWRKPLVRRWYGFVEMQKRV
ARQIPELLTVSSASASDILTDFAVSPEQLHVVPLGVDTKLFQPREGRVRNRIIAIASADVPL
KGVSHLLHAVARLRVERDVELQLVTKLEPNGPTEKLIAELGISDIVHTSSGLSDEELAAL
LASAEVACIPSLYEGFSLPAVEAMASGTPIVASRAGALPEVVGPDGECARLVTPADVDEL
TAVLGRLLDSPRELRRLGDNGRRRAVEVFSWQSVAAQTVAVYEKAIARVAAC

>ABK72422.1 Phosphatidylinositol α-mannosyltransferase (*Mycolicibacterium smegmatis*)

MRIGMVCPYSFDVPGGVQSHVLQLAEVLRDAGHEVSVLAPASPHVKLPDYVVSGGKA
VPIPYNGSVARLRFGPATHRKVKKWIAEGDFDVLHIHEPNAPSLSMLALQAAEGPIVATF
HTSTTKSLTLSVFQGILRPYHEKIIGRIAVSDLARRWQMEALGSDAVEIPNGVDVASFAD
APLLDGYPREGRTVLFLGRYDEPRKGMAVLLAALPKLVARFPDVEILIVGRGDEDELRE
QAGDLAGHLRFLGQVDDATKASAMRSADVYCAPHLGGESFGIVLVEAMAAGTAVVAS
DLDAFRRVLADGDAGRLVPVDDADGMAAALIGILEDDQLRAGYVARASERVHRYDWS
VVSAQIMRVYETVSGAGIKVQVSGAANRDETAGESV

>BAH69483.1 UDP-Glc - α-glucosyltransferase (*Mycoplasmopsis fermentans*)

MIWKFFVKKKGQRKMTNSEIRVILINDSFTPVIDGVVRVMQNYASNFIKKNVKVLVLAP
EYKKMKLDEDKGLNYSVIRIPRQLAKIGGYEVMNTILSRKIWKEIENFKPNIVHSHTPFY
AGDIANKIAKKFKIPSICSFHTLFYDSFLRFTKSKPIAKFLTHLMIKKLNKFDYIWPVSKV
AQKALIEYGYKKESEIVQLGTNFFYPENADELKNKVIKKWNINTNNKNLLYVSRLVWE
KNIKKVLLTYKKLVEENSNYHLTMVGGDINYKEIVKYAYKIGLKDKILFTGPIENQEELK
GIYLSHDLFFYPSLYETYGLVVRESAALKLPLLVVENTACAENVIDGQNSFICQDDVDN
MYEKIKQIFKDKNNLINVKNNMNNLFNSWEEAIEIVIEKYKKIIEHYKK

>CAA74303.1 CMP-NeuAc - α-galactoside-a-(2,6)-sialyltransferase - GT97 (*Neisseria meningitidis*)

MAVIIFVNGIRAVNGLVKSSINTANAFAEEGLDVHLINFVGNITGAEHLYPPFHLHPNVKT
SSIIDLFNDIPENVSCRNTPFYSIHQQFFKAEYSAHYKHVLMKIESLLSAEDSIIFTHPLQL
EMYRLANNDIKSKAKLIVQIHGNYMEEIHNYEILARNIDYVDYLQTVSDEMLEEMHSH
FKIKKDKLVFIPNITYPISLEKKEADFFIKDNEDIDNAQKFKRISIVGSIQPRKNQLDAIKII
NKIKNENYILQIYGKSINKDYFELIKKYIKDNKLQNRILFKGESSEQEIYENTDILIMTSES
EGFPYIFMEGMVYDIPIVVYDFKYGANDYSNYNENGCVFKTGDISGMAKKIIELLNNPE
KYKELVQYNHNRFLKEYAKDVVMAKYFTILPRSFNNVSLSSAFSRKELDEFQNITFSIED
SNDLAHIWNFELTNPAQNMNFFALVGKRKFPMDAHIQGTQCTIKIAHKKTGNLLSLLLK
KRNQLNLSRGYTLIAEDNSYEKYIGAISNKGNFEIIANKKSSLVTINKSTLELHEIPHELH
QNKLLIALPNMQTPLKITDDNLIPIQASIKLEKIGNTYYPCFLPSGIFNNICLDYGEESKIIN
FSKYSYKYIYDSIRHIEQHTDISDIIVCNVYSWELIRASVIESLMEFTGKWEKHFQTSPKID
YRFDHEGKRSMDDVFSEETFIMEFPRKNGIDKKTAAFQNIPNSIVMEYPQTNGYSMRSH
SLKSNVVAAKHFLEKLNKIKVDIKFKKHDLANIKKMNRIIYEHLGININIEAFLKPRLEKF
KREEKYFHDFFKRNNFKEVIFPSTYWNPGIICAAHKQGIKVSDIQYAAITPYHPAYFKSPK
SHYVADKLFLWSEYWNHELLPNPTREIGSGAAYWYALDDVRFSEKLNYDYIFLSQSRIS
SRLLSFAIEFALKNPQLQLLFSKHPDENIDLKNRIIPDNLIISTESSIQGINESRVAVGVYSTS
LFEALACGKQTFVVKYPGYEIMSNEIDSGLFFAVETPEEMLEKTSPNWVAVADIENQFFG
QEK

>CAD85125.1 Sucrose synthase (*Nitrosomonas europaea*)

MTTIDTLATCTQQNRDAVYTLLRRYFTANRTLLLQSDLREGLLQTEQDCGQSDMLRAF
VFRLQEGIFSSPWAYLALRPEIAKWEFMRIHQEHLIPEKLTISEFLKFKETVVKGEATESV
LEVDFGPFNRGFPRLKESRSIGQGVIFLNRKLSSEMFSRIEAGHTSLLHFLGVHAIEGQQL
MFSNNSHDIHAVRNQLRQALEMLETLDGTTPWIELAPKMNQLGFAPGWGHNANRVAE
TMNMLMDILEAPSPSALEEFLACIPMISRLLILSPHGYFGQDNVLGLPDTGGQVVYILDQ
VRALEKEMHDRLQLQGVQVEPKILIVTRLIPDAGDTTCNQRLEKVSGCTNTWILRVPFR
KHNGEIIPHWISRFEIWPHLEIFAGDVEREALAELGGHPDLIIGNYSDGNLVATLLSRRLG
VTQCNIAHALEKTKYLHSDIYWQENEDKYHFSCQYTADLLAMNSADFIVTSTYQEIAG
TREAEGQYESYQAFSMPDLYRVIHGIDLFDPKFNIVSPGANADIYFPYSDPNRRLHSLIPE
IESLIFDDATNLPARGYLQDPDKPLIFTMARLDRIKNITGLVELYAASPRLRSLANLVIVGG
KIDPQHSSDHEEQEQIHRMHQLMDEHELDQQVRWLGMRLDKNLAGELYRYIADKRGIF

VQPALFEAFGLTIIEAMASGLPTFATRYGGPLEIIQNNRSGFHIDPNQGAATADLIADFFEK
NLENPQEWERISQGALDRVASRYTWKLYAERMMTLSRIYGFWKFVSGLEREETDRYLN
MFYHLQFRPLANRLAHEI

>BAB75398.1 Sucrose synthase (*Nostoc* sp.)

MKILFLDQSGKPGGAELCLIDIAKPYRDRALVGLFADGAFKTLLEQHHIPVEVFTNQPIQ
VRKQSNLLQAFGSLGQLAPLVAKVVQTAHEYDLIYANTQKALVVGAIASFIARRPLVYH
LHDILSPEHFSQTNLRVAVNLANRFASLVIANSQASQTAFIQAGGRAELTKVIYNGFDINL
YKTSPSDISKLRQQLGVANNFVVGHFSRLSPWKGQHILIDALAQCPPQVTAILVGDALFG
EQDYVKELHQQITRLGLENRVKFLGFRADIPQLMAACDLVAHTSTAPEPFGRVIVEAML
CGKPVVAAKAGGAMELVEHGVNGFLTTPGESQELANIINTCIEDTQKTATIASNAQAIAS
QRFDVVTINQQIAETLSSLGFTR

>ABX31862.1 GDP-Man - glucosyl-3-phosphoglycerate 2-α-mannosyltransferase (*Petrotoga mobilis*)

MNNLYIFHYHYIKGGVSTVVRNIVKSLKDAYKITLFGSKKMGIDGIEEVLSYENVDFIDF
PELGYIYYDSTDYKTFLELKESIKNKLNNYHDERAIYWAHNYNLGKNPAFTEAFKEFITT
KNIPTIIQIHDFPECARWENYSFIRKFINSSLYPIRKNIQYATINLSDYNRLIKCGIPSENAFY
LPNAVEFAKNKDKIDDIDKDEVINKLKKLGYNVDPTNKNILYPTRTIRRKNILEAVLINRL
YGKSNLLVTLPANSDKERPYEKVVKETFESEKVKGAWAISAKDPSLFPYILNISDLFFSSS
VLEGFGMIYLESKFNEKNFLTRKLDVIEDFKNIKEISYYDRFLVSLSPKEINKVKEKYEEQ
INKIPISEENKNHLRQDLNNKFDKDLIDFSFLPVELQKKFCMEEEAKLNDLKEINKEIFDK
IEMLTSTNHIDQGINLEDFSLKAYKSKIFLLLDKVQARGNAPKGVQGSTKEIEDTIIDENIL
KSFLTIDNIRLLFSY

>CAM59608.1 α-xylosyltransferase (*Planktothrix agardhii*)

MKIAFFDYIWTLAEPPKFGSGILCYQLALRLASDNDLIFYGSKAAHHQELEYSEDGITYR
RIPNNFIDKILGLLYVLDDWKIFPAKRPFFTSILSDFKYYWQIAKDLQSQNCDIIQINFAFH
VSPLIRAFNPQAKIVIFLQTEWLTQLDPKMIERKLKAVDLVIGCSDYITNQIRTRFPNVPC
QTIFNGVDINHFVVNNEAKKNQKKEMKTLLFVGRISPEKGLHILLEAFNKVILQYPQVQ
LKLIGPEGKYGVLPIQMINTEDPYFQTLIPFYQGEYSDQLRQIISPNAANSVCFFGPVEQL
DLVEHYQDADIFIFPSVWNEPFGIPLVEAMAMELPVIATYSGAFPEIVEDEKTGLLVERSN
PDALAEAILRLLSDENLSQEMGKAGRQRVVDKFSWEQISETLLVEFHKICDPTC

>QKR24265.1 N-acetylgalactosaminyltransferase (*Pseudomonas aeruginosa*)

MTLAFILYKYFPFGGLQRDFMRIALECQRRGHDIRVYTLIWEGDVPDGFEVLVAPVRSIF
NHRRNEKFTAWVRADLARRPVQRVIGFNKMPGLDVYYAADACFEEKAQTLRNPLYRQ
WGRYRHFAGYERAVFDPASKTEILMISEVQQPLFVKHYGTQAERFHLLPPGISQDRRAP
ANAADVRAEFRREFGLEEDDLLLVQIGSGFKTKGLDRSLKALSALPKALRRRTRLIAIGQ
DDPKPFLLQIAALGLNDQVQILKGRSDIPRFLLGADLLIHPAYNENTGTVLLEALVSGLP

VLVTDVCGYAHYIAEADAGRVLPSPFEQDSLNRLLAEMLEDAPARAAWSRNGLAYADH
ADLYSMPQRAADLILGEAS

>AAC05215.1 GDP-Man - α-mannosyltransferase (*Rhizobium leguminosarum* bv. *viciae*)

MPDIRDVEIIAPNFKRRLSGVTSTIVQLIPCQIRLGIKIATLGPGLPEDLPKLKWRQLLGLW
RPPARRRRRVWHARRNNEMAVGILLRHLTRMPLKLLFTSAAQRRHTAYTKWLIRRMDA
VIATSDRSGSFLEVPHTVIQHGVDLALFHPPEAAEDGIAATGLPGRHLVGCFGRVRHQKG
TDLFVRAMIELLPQHTEWTAVVSGRVTAEHVAFADKLKADVVAAGLSDRILFLGEVPDI
KIWYRRLTLYVAPSRNEGFGLTPLEAMASRTAVVASDAGAYAELIVTGETGSVVAASDGE
ALTRAIAPYIADPALAVAHGENALRHVRANFALEREASAIGAVYNSLLGDNRS

>ABG05883.1 NDP-Glc - α-glucose α-glucosyltransferase / a,α-trehalose synthase (*Rubrobacter xylanophilus*)

MLQRVNPGHKALADYRSIIRRELYGELQELAGRLRGARVLHINATSFGGGVAEILYTLVP
LARDAGLEVEWAIMFGAEPFFNVTKRFHNALQGADYELTIEDRAIYEEYNRRTAQALAE
SGEEWDIVFVHDPQPALVREFSGGLGEGTRWIWRCHIDTSTPNRQVLDYLWPYIADYDA
QVYTMREYTPPGVEMPGLTLIPPAIDPLSPKNMALSRDDASYIVSQFGVDVERPFLLQVS
RFDPWKDPLGVIDVYRMVKEEVGEVQLVLVGSMAHDDPEGWDYWYKTVNYAGGDP
DIFLFSNLTNVGAIEVNAFQSLADVVIQKSIREGFGLVVSEALWKARPVVASRVGGIPMQI
TAGGGILIDTIPEAAAACAKLLSDPEFAREMGRRGKEHVRANFLTPRLLRDDLRLFAKLL
GV

>AAL22573.1 UDP-GlcNAc - LPS α-N-acetylglucosaminyltransferase (*Salmonella enterica subsp. enterica*)

MIKKIIFTVTPIFSIPPRGAAAVETWIYQVAKRLSIPNAIACIKNAGYPEYNKINDNCDIHYI
GFSKVYKRLFQKWTRLDPLPYSQRILNIRDKVTTQEDSVIVIHNSMKLYRQIRERNPNA
KLVMHMHNAFEPELPDNDAKIIVPSQFLKAFYEERLPAAAVSIVPNGFCAETYKRNPQD
NLRQQLNIAEDATVLLYAGRISPDKGILLLLQAFKQLRTLRSNIKLVVVGDPYASRKGEK
AEYQKKVLDAAKEIGTDCIMAGGQSPDQMHNFYHIADLVIVPSQVEEAFCMVAVEAMA
AGKAVLASKKGGISEFVLDGITGYHLAEPMSSDSIINDINRALADKERHQIAEKAKSLVF
SKYSWENVAQRFEEQMKNWFDK

>AAC60480.2 LPS/UndPP-GlcNAc α-1,3-galactosyltransferase (*Shigella dysenteriae*)

MKISIIGNTANAMILFRLDLIKTLTNKGISVYAFATDYNDSSKEIIKKAGAIPVDYNLSRSG
INLAGDLWNTYLLSKKLKKIKPDAILSFFSKPSIFGSLAGIFSGVKNNNAMLEGLGFLFTE
QPHGTPLKTKLLKNIQVLLYKIIFPHINSLILLNKDDYHDLIDKYKIKLKSCHILGGIGLD
MNNYCKSTPPTNEISFIFIARLLAEKGVNEFVAAAKKIKKTHPNVEFIILGAIDKENPGGL
SESDVDTLIKSGVISYPGFVSNVADWIEKSSVFVLPSYYREGVPRSTQEAMAMGRPILTT
NLPGCKETIIDGVNGYVVKKWSHEDLAEKMLKLINNPEKIISMGEESYKLARERFDANV
NNVKLLKILGIPD

>EHM57698.1 UDP-GlcNAc - α-1,4-N-acetylglucosaminyltransferase (*Staphylococcus aureus subsp. aureus*)

MKKIFMMVHELDVNKGGMTSSMFNRSKEFYDADIPADIVTFDYKGNYDEIIKALKKQG
KMDRRTKMYNVFEYFKQISNNKHFKSNKLLYKHISERLKNTIEIEESKGISRYFDITTGT
YIAYIRKSKSEKVIDFFKDNKRIERFSFIDNKVHMKETFNVDNKVCYQVFYDEKGYPYIS
RNINANNGAVGKTYVLVNKKEFKNNLALCVYYLEKLIKDSKDSIMICDGPGSFPKMFN
TNHKNAQKYGVIHVNHHENFDDTGAFKKSEKYIIENANKINGVIVLTEAQRLDILNQFD
VENIFTISNFVKIHNAPKHFQTEKIVGHISRMVPTKRIDLLIEVAELVVKKDNAVKFHIYG
EGSVKDKIAKMIEDKNLERNVFLKGYTTTPQKCLEDFKLVVSTSQYEGQGLSMIEAMIS
KRPVVAFDIKYGPSDFIEDNKNGYLIENHNINDMADKILQLVNNDVLAAEFGSKARENII
EKYSTESILEKWLNLFNS

>QDA62746.1 UDP-Glc - Teichoic acid α-glucosyltransferase (*Staphylococcus epidermidis*)

MKQTYMIVNELDVNKGGMTTAMLTRSKFFLDNEISGDIITFDFKANYKDILKELVQSKK
MDKRTQMHNPFIYFKNISNLQHKKYNYTMTRNLSNLLKDSVEIKENSRISRFFNIMSGE
YLAYKRETEQETIFDLFKNNLRYKRIYFYKGKIVKTEVFNSDNNLIAEQFYDDNGYLYL
YRQINPEKKSIGKTYLVCKEKQFKNNVEFCSYFLDKLIPDINDNIIICDGPGSFPKILKTNH
KNVKKFAVIHVNHYKNFDDTGAVKKQEDYILRNANKINGVVMLTEAQKKDIIEKYKIT
NAYVISNFINITDDYRDKNDNKVVGHISRLVPQKGLPYLIDVAKKVVEQDNSVEFHLYG
TGEEKSKIENLIQESNLTNNVKLLGYTTNAIEKIKDFRCVISTSQFEGQGLSLIEAMLLKK
PVVAFDVKYGPSDFVKDGKNGYLIENKDIKKMANKILKLLHDKELSKSLGKHGRDTIID
MYQPEKLMVKWKQLFN

>AAK17002.2 UDP-GlcNAc - α-N-acetylglucosaminyltransferase (*Streptococcus gordonii*)

MTVYNINLGIGWASSGVEYAQAYRAQILRRIQQPAKFIFMDMILADNIQHLTENIGFLDE
EIIWLYNYFTDIKIAPTTVTLDQVLAQVAGQPERSEKEGKIVRYFYPQDDQFITCYLRQE
DQDSVEHVEYVSRGRLIRKDYFSYVRYASEYFAPHNDAATLYQRRFYHEDGSVAYDMLI
EDGQEKLYRFPDRIFYSKAELVRYFLQCLQLQADDVVILDRETGIGQVVFEESQKAKLG
VVVHAEHFSENASSDDYILWNNFYDYQFTNADKVDFFIVATEAQKRILEQQFQHYSDK
QPQIATIPVGSLDQLTYPKEPRKPYSMITASRLATEKHIDWLVAATVQAHAQLPELTLDIY
GKGSEEDKLRRRIEEAGAQDYIRLKGHADLSQIYAGYELYLTASTSEGFGLTLMEAVGSG
LPLIGFDVRYGNQTFIDDGKNGYLLPVSSNHVEDQIIAAFVEKIIALFSQGRQQEMSQHS
YQVAENYLTSRVEAAWTQLLKEVRDDSAL

>CAI34028.1 UDP-β-L-Rha - β-glucose β-1,4-rhamnosyltransferase (*Streptococcus pneumoniae*)

MKKSVYIIGSKGIPAKYGGFETFVEKLTAFQQDKAIQYYVACMRENSAKSGITEDVFEH
NGAICYNVDVPNIGPARAIAYDIAAINRAIEIAKENKDEDPIFYILACRIGPFIHGIKKRIQA
IGGALLVNPDGHEWLREKWSLPVRKYWKYSERLMVKHADLLVCDSKNIEKYIQEDYK
QYQPKTTYIAYGTDTTRSILKSSDEKVRSWFKEKNVSENEYYLVVGRFVPENNYEAMIR

GFLASNSKKDFVLITNVEQNKFYNQLLAKTGFDKDPRVKFVGTVYEQELLKYIRENAFA
YFHGHEVGGTNPSLLEALASTKLNLLLDVGFNREVGEQSAIYWKKDELSQVIEKVEQF
DAKMIDELDRQSNQRIVDFFTWEKIVTDYEKLFKK

>AAC44013.1 EPS α-1,6-galactosyltransferase (*Streptococcus thermophilus*)

MNEQVTFILCDFLVREIKPKYDLLAYQFISKKIKEIKPDIVHCHSSKAGVIGRLAAKRRGV
KKIFYTPHAYSFLAPEFSGKKKFLFVQIEKFLSRFATTKIFCVSIAEMQAALEVNLDKTDK
FQVIYNGLPEIDLPSKETIRAQLGLEKAAVVIGNNAKMSEQKNPMFFMEIARKMIRQNA
NWHFVWVGDGQLMPLFQSFIKQNGLEGNIHLLGERPDSEIVVTAYDIFLTTSQYEGLPY
APIEAMRAGVPILATKVVGNSELVIEGKNGYLIDLEWSKSVEEKLYKAAKIDAQMIKAD
FRQRFAIDQILKQIETIYLA

>BAD95821.1 UDP-GlcNAc - 2-deoxystreptamine O-α-N-acetylglucosaminyltransferase (*Streptomyces fradiae*)

MRVLRLTPFFHHDCVTAWPAEFDAVGGMQVQILRLSRELADRGVEQLVMTVGFPGLPR
ERVDRPGLRVRVTRAPLPRLRSELTGLVGLNQAWLAAVLTACAPLRRTWRPDLVHVHA
DGQLWALLAGPLVSRLVGAPYCLTLHCSRLASYEPMSRFDRLQHRLVAAAERYALRRA
RRVSTLTSRTADTVARLLPLDRALVDVLPDSVGDVRPVARPEAEEYVRSLGVPAGRPVV
GWVGRVAHEKGWRDFVAMAERWDAGSGAPGAVFAVVGDGPQRERMREAVEAAGLA
DRFVFTGFLPHDAVPSVMTALDVLVMPSAHEELGGSALEAMVCGTPVAGYAVGGLRDT
VGSVTPSLLVPRGDVAALTRAAGDAVTDAERHRKTVAAAVPDLLGRYGADTVERALEH
YRLAVGRASGGGAGWAP

>BAD20768.1 UDP-GlcNAc - 2-deoxystreptamine O-α-N-acetylglucosaminyltransferase (*Streptomyces kanamyceticus*)

MRVLRLTPFYHHDCVDSWPAEFDAVGGMQVQILRMSRALAELGVRQQVLTVGFPGLPR
VRRDSENLVVRITRAPLPRLRSRITGLVGLNQAWLAAALTECVKLRRRWPADLIQVHLD
GQLWALLAGPVAARLVGVPYTVTVHCSRLAVYQPMSTVDRIQHPLVTAVERWALRRAA
GITTLTERTATVLAAELGAAQRVIDVVPDAVDPDRAEAAPAEVERLKKRFGLPQEGGPVI
GFVGRIAHEKGWRHAVQAVAELADAGRDFTFLVVGDGPQRADMEAAVAEAGLTDRFV
FTGFLPNDEIPAVMTALDVLLMPSVHEELGGSAVEAMLAGTPVAAYGVGGLCDTVGKV
TPSLLAAPGQVAELARTVKRVLDDPAPVLAELRAGREWLADEFGVHHAAGLALAHYE
RVLGKER

>AAK70860.1 UDP-glucose - Tetrahydrobiopterin glucosyltransferase (*Synechococcus elongatus*)

MTAHRFLFVSTPVGPLGSGRGGGVELTLPNLAKALTQRGHQVSVLAPAGSVLPDLPLET
VPGTWQSTAQSHGRATPAEIPAESVLARLWDRAHQQQADFDLILNFAYDWLPLYLTPFF
KTPVAHLISMGSLSEVMDQAIATSLDRYPGSIAVHSLAQAATFPFGDRCLCIGNALDLAA
YGFNPEPEPVLGWVGRIAPEKGLEDAIQAAQQAGLPLRVWGALTEPDYWQRLQQQFG
DRAVSYQGFVSTDELQRGLGRCQGLLMTPKWVEAFGNVAIEALACGLPVIAYARGGPL

EIIEQGKSGWLVEPDQQAALVNAIGQLSSLDRAYCRAQAEARFSLAAMGQRLEAWLLP
LLSRARGF

>BAA10782.1 Sucrose-phosphate synthase (*Synechocystis* sp.)

MSYSSKYILLISVHGLIRGENLELGRDADTGGQTKYVLELARALVKNPQVARVDLLTRLI
KDPKVDADYAQPRELIGDRAQIVRIECGPEEYIAKEMLWDYLDNFADHALDYLKEQPE
LPDVIHSHYADAGYVGTRLSHQLGIPLVHTGHSLGRSKRTRLLLSGIKADEIESRYNMAR
RINAEEETLGSAARVITSTHQEIAEQYAQYDYYQPDQMLVIPPGTDLEKFYPPKGNEWE
TPIVQELQRFLRHPRKPIILALSRPDPRKNIHKLIAAYGQSPQLQAQANLVIVAGNRDDIT
DLDQGPREVLTDLLLTIDRYDLYGKVAYPKQNQAEDVYALFRLTALSQGVFINPALTEPF
GLTLIEAAACGVPIVATEDGGPVDIIKNCQNGYLINPLDEVDIADKLLKVLNDKQQWQF
LSESGLEGVKRHYSWPSHVESYLEAINALTQQTSVLKRSDLKRRRTLYYNGALVTSLDQ
NLLGALQGGLPGDRQTLDELLEVLYQHRKNVGFCIATGRRLDSVLKILREYRIPQPDMLI
TSMGTEIYSSPDLIPDQSWRNHIDYLWNRNAIVRILGELPGLALQPKEELSAYKISYFYD
AAIAPNLEEIRQLLHKGEQTVNTIISFGQFLDILPIRASKGYAVRWLSQQWNIPLEHVFTA
GGSGADEDMMRGNTLSVVVANRHHEELSNLGEIEPIYFSEKRYAAGILDGLAHYRFFEL
LDPV

>BAC09142.1 Sucrose-phosphate synthase (*Thermosynechococcus vestitus*)

MQALSTRTAKNTLPRAFAMPQPQVPARQPIALISVHGDPAADVGHESAGGQNIYVRQLG
EALAAAGWHVDMFTRKTDPNDPDVIEHSPHCRTIRLQAGPLTYIPREKLFETLPKFVEAF
KAYHAKYGYPLIHTNYWLSGWVGWQLRQQFNFQWLHTYHSLGVVKYQVASEQAQR
DETRLMVEKAILENADCVIVTSPQEEAYLRRWVSKAGQTRLIPCGTNLKLFYPVADARA
QLNLPADEPIVLYVGRFDRRKGIETLVAAMAQIPQGQLLLVGGSDPQRSDGAERRRIEGL
VQEYNLGDRVTFVGQIDHEYLAVYYSAANVCVVPSYYEPFGLVAIEAMACGTPVIASAV
GGLQFTVIPEETGLLVPPQDANALANAIQRILADPAWARTLGKNGRERVQALFNWEAIA
LQMGQLYRQLFAASLMGNSPRLEMVKNTASLAAVTKAALAS

>AAD35825.1 UDP-Glc - 1,2-diacylglycerol 3-glucosyltransferase (*Thermotoga maritima*)

MNIAMFSDTYAPQINGVATSIRVYKKKLTERGHKVVVVAPSAPEEEKDVFVVRSIPFPFE
PQHRISIASTKNILEFMRENNVQIIHSHSPFFIGFKALRVQEEMGLPHVHTYHTLLPEYRH
YIPKPFTPPKRLVEHFSAWFCNMTNVVIAPTEDIKRELESYGVKRPIEVLPTGIEVEKFEV
EAPEELKRKWNPEGKKVVLYAGRIAKEKNLDFLLRVFESLNAPGIAFIMVGDGPEREEV
EEFAKEKGLDLKITGFVPHDEIPLYYKLGDVFVFASKTETQGLVLLEALASGLPVVALK
WKGVKDVLKNCEAAVLIEEENERLFAEKIKHILKNDRLREELSTKGREFVRKEWSVDRF
VQRLEEIYTRAIEEGPVEINTSLMIKEFVKFEKLKEFFSKIEDRIWR

>AAA86376.1 GDP-Man - cellobiosyl-PP-polyprenyl α-1,3-mannosyltransferase (*Xanthomonas campestris*)

MKVVHVVRQFHPSIGGMEEVVLNVARQHQANSADTVEIVTLDRVFTDPSAQLAQHEL
HQGLSITRIGYRGSSRYPIAPSVLGAIRSADVVHLHGIDFFYDYLALTKPLHGKPMVVST

HGGFFHTAYASRMKQIWFQTLTRTSALAYARVIATSENDGDLFAKVVAPSRLRVIENGVD
VEKYAGQGARAPGRTMLYFGRWSVNKGLIETLELLQAALTRDPQWRLIIAGREYDLNE
ADLRKAIAERGLQDKVQLSMSPSQQQLCALMQQAQFFVCLSRHEGFGIAAVEAMSAGL
IPILSDIPPFVRLATESGQGVIVNRDRIQAAADSVQALALQANADFDARRTATMAYVARY
DWRHVVGRYIDEYHAALGTPRTQEAVR

>AAF85163.1 GDP-Man - cellobiosyl-PP-polyprenyl α-1,3-mannosyltransferase (*Xylella fastidiosa*)

MKVVHVVRQFHPSIGGMEDVVFNIAMQLHLHAGIDVDVVTLNRVFTQSDVLLPCTDK
YQGVSIQRIGYRGSSRYPLAPWVLRMLDKADVIHVHGIDFFYDFLALTRVLHGKPMVV
STHGGFFHTDYASRLKLLWFNTLTRLSALAYARIIASSESDGALFSKIVAPSRLRVIENGV
DVEKYARCGASEAGRTLLYFGRWSMNKGLLETLQLLAVLYVLDPRWRLIIAGREYDYD
QAALAYEVDRLGLSEQVHFHCSPSQSQLRFLMEQAQFFISLSRHEGFGIAAVEAMSAGLI
PVLSDIPPFARLHRESGLGVLVDPLQPQQAAVAVQGLAVQVDTHFIDWRSQAMAFSDRY
HWRYVIGCYQDEYCRALGLGGEQEFLR

>QAT46148.1 UDP-Glc - steviol β-glucosyltransferase - GT1 (*Bacillus subtilis*)

MANVLMIGFPGEGHINPSIGVMKELKSRGENITYYAVKEYKEKITALDIEFREYHDFRGD
YFGKNATGDEERDFTEMLCAFLKACKDIATHIYEEVKHESYDYVIYDHHLLAGKVIAN
MLKLPRFSLCTTFAMNEEFAKEMMGAYMKGSLEDSPHYESYQQLAETLNADFQAEIKK
PFDVFLADGDLTIVFTSRGFQPLAEQFGERYVFVGPSITERAGNNDFPFDQIDNENVLFIS
MGTIFNNQKQFFNQCLEVCKDFDGKVVLSIGKHIKTSELNDIPENFIVRPYVPQLEILKR
ASLFVTHGGMNSTSEGLYFETPLVVIPMGGDQFVVADQVEKVGAGKVLKKEELSESLL
KETIQEVMNNRSYAEKAKEIGQSLKAAGGSKKAADSILEAVKQKTQSANA

>CAB14063.1 UDP-Glc: glycocin S-β-glucosyltransferase - GT2 (*Bacillus subtilis subsp. subtilis*)

MKLSDIYLELKKGYADSLLYSDLSLLVNIMEYEKDIDVMSIQSLVAGYEKSDTPTITCGII
VYNESKRIKKCLNSVKDDFNEIIVLDSYSTDDTVDIIKCDFPDVEIKYEKWKNDFSYARN
KIIEYATSEWIYFIDADNLYSKENKGKIAKVARVLEFFSIDCVVSPYIEEYTGHLYSDTRR
MFRLNGKVKFHGKVHEEPMNYNHSLPFNFIVNLKVYHNGYNPSENNIKSKTRRNINLT
EEMLRLEPENPKWLFFFGRELHLLDKDEEAIDYLKKSINNYKKFNDQRHFIDALVLLCT
LLLQRNNYVDLTLYLDILETEYPRCVDVDYFRSAILLVDMQNKLTSLSNMIDEALTDERY
SAINTTKDHFKRILISLNIQLENWERVKEISGEIKNDNMKKEIKQYLANSLHNIEHVLKGI
EV

>sp|O93869| UDP-Glc glycogen synthase - GT3 (*Neurospora crassa*)

MAHDNREPREVKNHLLFEVATEVAHRVGGIYSVLKSKAPVTTAEYGDRYTLIGPLNHQS
AAVEVEELEPSNPELKATIQAMRDRGIGILYGRWLIEGAPRVLLFDTKTAYGYMNEWKT
DLWNVASIPSPDNDEETNEAIVFGYLVAWFLGEFVCHEKRKAVIAHFHEWLAGVALPLT
KKRQIDVTTIFTTHATLLGRYLCAGSVDFYNNLQWFDVDAEAGKRGIYHRYCIERAAA

HSCDVFTTVSHITAYESEHLLKRKPDGVLPNGLNVTKFSAMHEFQNLHQQNKEKIHDFV
RGHFYGHYDFEPENTLYFFTAGRYEFRNKGVDMFIESLARLNHRLKTAGSKTTVVAFII
MPAQTTSLTVEALKGQAVIKSLRDTVDVIERGIGRRIFERSVKWHEGDPLPEEKELITSQ
DRVLLRRRLFAMKRHTLPPIVTHNMLNDHEDPILNQIRRVQLFNHPSDRVKIVFHPEFLS
SANPVLPLDYDDFVRGTHLGVFASYYEPWGYTPAECTVMGVPSITTNLSGFGCYMEELI
ENSSDYGIYIVDRRSKGVDDSVNQLTQYMFEFTQKSRRQRINQRNRTERLSDLLDWKR
MGMEYVKARQLALRRAYPTSFNGEEEEDFIPGVEQKISRPFSVPGSPRDRTGMMTPGDF
ASLQESHEGLSTEDYVAWKLPEEEDPEEYPFPLTLKQRTGPGSPLDSIQGLQLNGTR

>AAC00217.1 ADP-Glc glycogen synthase - GT5 (*Bacillus subtilis*)

MKILFAVSECTPFVKSGGLADVAGALPKALARLGNEVAVMLPKYSQIPEPWKKRMKKQ
AECTVAVGWRQQYCGIEHMAENDVNYYFIDNEYYFNRDSLYGHYDDGERFAFFSRAVL
EAAKVVNVQADIVHTHDWHTAMVNYLLKEEYRKHPFYERMKSVLTIHNLQFQGIFPPD
VTHDLLGLEMDHFHYERLECNGFVNFMKAGIIAADHVTTVSPTYRNEIMTPYYGEQLE
QVLQYREDDVTGILNGIDDTFYQPKSDPYIEAQYDSGDLACKLENKTKLQQRMGLPEK
NDIPLISMVTRLTKQKGLDLVRRIMHELLEEQDIQLVVLGTGEREFEDYFRYAEFAFHEK
CRAYIGFDEPLAHQIYAGSDMFLMPSKFEPCGLGQLIALQYGAIPIVRETGGLYDTVRAY
QEEEGTGNGFTFSAFNAHDLKFTIERALSFYCQQDVWKSIVKTAMNADYSWGKSAKEY
QRIFEQVTRSGRDVLE

>AAV80756.1 N-acetyllactoside 3-α-galactosyltransferase - GT6 (*Escherichia coli*)

MVINIFYICTGEYKRFFDKFYLSCEDKFIPEFGKKYYVFTDSDRIYFSKYLNVEVINVEK
NCWPLNTLLRFSYFLKVIDKLQTNSYTFFFNANAVIVKEIPFSTFMESDLIGVIHPGYKNR
ISILYPWERRKNATCYLGYLKKGIYYQGCFNGGKTASFKRLIQICNMMTMADLKKNLIA
KVHDESYLNYYYYYNKPLLLSELYSWPEKYGENKDAKIIMRDKERESWYGNIKK

>tr|D3UIY4| Glycoprotein-fucosylgalactoside α-N-acetylgalactosaminyltransferase - GT6 (*Helicobacter mustelae*)

MQSTAQNTQQNTHFAGSSQTTPQAAQSVQQASLALPKSSPTCYKIAILYICTGAYSIFWQ
DFYDSAKVHLLPAHRLTYFVFTDADSLYAEEASDVRKIYQENLGWPFNTLKRFEMFLG
QEEALREFDFVFFFNANCLFFQHIGDEFLPIEEDILVTQHYGFRDASPECFTYERNPKSLA
YVPFGKGKAYVYGSTNGGKAGAFLALARTLQERIQEDLSRGIIAIWHDESHLNAYIIDHP
NYKMLDYGYGFPEGYGRVPGGGVYIFLRDKSRVIDVNAIKGMGSPANRRLKNALRKLK
HFSKRLLGR

>AAG23384.1 UDP-GalNAc - β-1,4-N-acetylgalactosaminyltransferase - GT7 (*Caenorhabditis elegans*)

MAFRHLAVARLKSLLVLCAVLLLVHAMIYKIPSLYENLTIGSSTLIADVDAMEAVLGNTA
STSDDLLDTWNSTFSPISEVNQTSFMEDIRPILFPDNQTLQFCNQTPPHLVGPIRVFLDEPD
FKTLEKIYPDTHAGGHGMPKDCVARHRVAIIVPYRDREAHLRIMLHNLHSLLAKQQLD
YAIFIVEQVANQTFNRGKLMNVGYDVASRLYPWQCFIFHDVDLLPEDDRNLYTCPIQPR

HMSVAIDKFNYKLPYSAIFGGISALTKDHLKKINGFSNDFWGWGGEDDDLATRTSMAG
LKVSRYPTQIARYKMIKHSTEATNPVNKCRYKIMGQTKRRWTRDGLSNLKYKLVNLEL
KPLYTRAVVDLLEKDCRRELRRDFPTCF

>AAC16411.1 LPS α-1,2-glucosyltransferase - GT8 (*Salmonella enterica subsp. enterica serovar typhimurium*)

MDSFPEIEIAEYKVFDESNNNDDNVLNISYGVDENYLDGVGVSIASVVLNNNIPLAFHII
CDSYSPCFVKYIERLAVQHHIKISLYLIKVESLEVLPQTKVWSRAMYFRLFAFDYLSKKV
NTLLYLDADVVCKGSLQDLLQLDLTEKIAAVVKDVDSIQNKVNERLSAFNLQGGYFNS
GVVFVNLKLWKENALTKKAFLLLAGKEADSFKYPDQDVLNILLQDKVIFLPRPYNTIYT
IKSELKDKSHKKYSNIINDNTILIHYTGATKPWHAWANYPSVIYYKNARLNSPWKDFPA
KDARTIVEFKKRYKHLLVQGHYFKGLLAGSAYLYRKLFHK

>AAC76647.1 LPS β-N-acetylglucosaminyltransferase - GT9 (*Escherichia coli*)

MRLGTFHKKKRFYINKIKINFLSFLFRNKINNQITDPAQVKSCLIIHDNNKLGDLIVLSSIY
RELYSKGVKITLLTNRKGGEFLSNNKNIFEFCIKESTGFLEMLTLCKHLRDLQFDIVLDPF
ETMPSFKHSLILSSLKDSYILGFDHWYKRYYSFYHPHDECLKEHMSTRAIEILKHIYGEG
KFSTNYDLHLPVDVEDKIKEFIGDTRIVIINPLGAKKICRLTFEQIKVIYQEVKTHFENYRI
IFTGLPQDLLTIPILEIETLPFDEFIYTVALTKYSDFVISVDTALVHIAAAYHKPTLAFYPNS
RTPEYPSHLIWSPNHHKSIQIVSPTYTVKDIDTETLTNSVKRLSCIDKK

>AAD06573.1 4-galactosyl-N-acetylglucoside 3-α-L-fucosyltransferase - GT10 (*Helicobacter pylori*)

MFQPLLDAFIESTPIKKKITFKSPPPPLKIAVANWWGGAEEFKKSTLYFILSQRYTITLHQN
PNEPSDLVLGSPIGSARKILSYQNTKRVFYTGENEVPNFNLFDYAIGFDELDFRDRYLRM
PLYYASLHYKAESVNDTTAPYKLKDNSLYALKKPSHHFKENHPNLCAVVNDESDPLKR
GFASFVASNPNAPIRNAFYDALNSIEPVTGGGSVKNTLGYNVKNKSEFLSQYKFNLCFE
NTQGYGYVTEKIIDAYFSHTIPIYWGSPSVAKDFNPKSFVNVCDFKNFDEAIDYVRYLHT
HPNAYLDMLYENPLNTLDGKAYFYQNLSFKKILDFFKTILENDTIYHDNPFIFYRDLNEP
LVAIDDLRVNYDDLRVNYDDLRVNYDDLRVNYDRLLQNASPLLELSQNTTFKIYRKAY
QKSLPLLRAIRRWVKKLGL

>KSZ46701.1 LafA (*Listeria monocytogenes*)

MNIGIFTDTYSPQISGVATSIMIMENELRKQGHTVYIFTTTDPNADRESEEGRVFRLPSIPF
VFFPERRVAIAGMNKFIKLVGRLDLDIIHTHTEFSLGLLGKRIAKKYHIPSIHTYHTMYVD
YLHYIAKGKILTPSMVGKMTKSFCDSYDAIITPTAKVRHHLEEQGIHKLMYTVPTGTDIS
SFAPVEKQRILDLKKLLGIGENDPVILSLGRIAHEKNIDAIINAMPEVLQTKTTAKLVIVG
DGPVRKDLEKLVEEKQLADHVIFTGAVDWENISLYYQLGDLFVSASTTETQGLTYAEAM
AASLPVVAKRDESIEGFLSDRETAFLFNEDDELASLLINILSDKNTATLVATNGRVKVESIS
ADQFGINIESTYNEVREIYRVKRQNGTIKVKPTLIKSKIASQVFSLSSSTHVQRKERSSRR
D

>QHN44351.1 LafA (*Salmonella* sp.)

MLSINTNNASMAAVNAISKSSSSLSTSMERLATGNRINSSADDAAGKQIANRLTAQSSG
MGVALSNINDATAMLQTADSMFDEMSDVLGRMKDLSTQAANGTYSDDDLQAMQDEY
DELGQQMSDMLQNTTYGGTNLFGVSGTSNTGTDGLFQSAVTFQVGAESSDTMTVNISS
QLNTLVTDLSAISNSFSADQADTTGTAGVSGGTELTASGSANQMITSISTAMDDVSQIQS
KLGASINRLNDTANNLTSMQDNTEVAIGNIMDTDYATEASNMTKQQVLMQTGITMLKQ
SNSMSSMVSSLLQ

>KAJ82916.1 LafB (*Enterococcus faecalis*)

MKILLYFEGEKILAKSGIGRALDHQKRALSEVGIEYTLDADCSDYDILHINTYGVNSHR
MVRKARKLGKKVIYHAHSTEEDFRNSFIGSNQLAPLVKKYLISLYSKADHLITPTPYSKT
LLEGYGIKVPISAISNGIDLSRFYPSEEKEQKFREYFKIDEEKKVIICVGLFFERKGITDFIE
VARQLPEYQFIWFGDTPMYSIPKNIRQLVKEDHPENVIFPGYIKGDVIEGAYAAANLFFFP
SREETEGIVVLEALASQQQVLVRDIPVYQGWLVANENCYMGHSIEEFKKYIEGLLEGEIP
STREAGYQVAEQRSIKQIGYELKEVYETVLS

>KAJ82915.1 LafA (*Enterococcus faecalis*)

MKIGFFTDTYFPQVSGVATSIKTLKDELEKHGHEVYIFTTTDPNATDFEEDVIRMPSVPFV
SFKDRRVVVRGMWYAYLIAKELELDLIHTHTEFGAGILGKMVGKKMKIPVIHTYHTMY
EDYLHYIAKGKVVRPSHVKFFSRVFTNHTTGVVCPSERVIEKLRDYGVTAPMRIIPTGIEI
DKFLRPDITEEMIAGMRQQLGIEEQQIMLLSLSRISYEKNIQAIIQGLPQIIEKLPQTRLVIV
GNGPYLEDLKELAEELEVSEYVQFTGEVPNEEVAIYYKAADYFVSASTSETQGLTYTEA
MAAGVQCVAEGNAYLNNLFDHESLGKTFKTDSDFASTLIDYIQANIKMDQTILDEKLFEI
SSTNFGNKMIEFYQDTLIYFDQLQMEKENADSIKKIKVKFTSLRK

>WP_009924347.1 LafB (*Listeria monocytogenes*)

MIKLTMLSSAEKVKGQGVASAYRELVNLLEERYKNEIDMKINSFEKSDITHYHTVDFRFF
LSTFFKKKRGVRVGYVHFLPETMEGSLKLPWIARVVFYKYLIGFYKRMDEIVVVNPSFI
PKLTAYNIPEEKIHYIPNFVSKKSFFPISKTEKELAREKYGIPADKFTVIGIGQVQHRKGVL
DFIEVAKQLPDVQFVWAGGFSFGKITSGYEELKKIYDNPPNNVNFIGIVDRSEMNTCINM
ADVFFMPSYNELFPMAILEAMSSDVPILLRNLDLYEEILDGYYVKEVDNPGFIRAIERLE
NDTNYYNEMLQAAKRGAAYYSEDRLAEIWLGFYQGLLTKE

Figure S2. Amino acid sequences of *Ef*LafB and related glycosyltransferases for phylogenetic analysis.

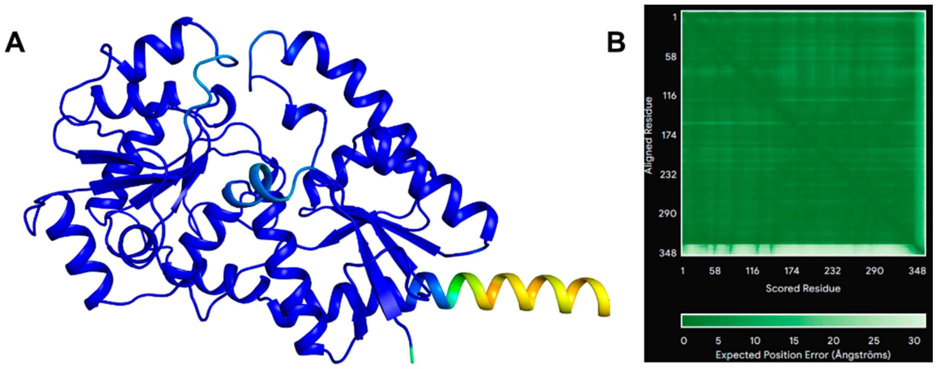

Figure S3. Structural model of *Ef*LafB predicted by AlphaFold and its confidence assessment. (A) Cartoon representation of the predicted structure of *Ef*LafB. The model exhibits a characteristic GT-B fold, with two α/β domains. The confidence of the structural prediction is color-coded, with regions in dark blue indicating high reliability (pLDDT >90), while regions with lower confidence are shown in yellow to green. (B) Expected Position Error (Å) plot from AlphaFold3 server, illustrating the estimated accuracy of residue positioning in the model. Dark green areas correspond to high-confidence regions with minimal positional error, whereas lighter regions indicate lower confidence, particularly in flexible or unstructured regions.

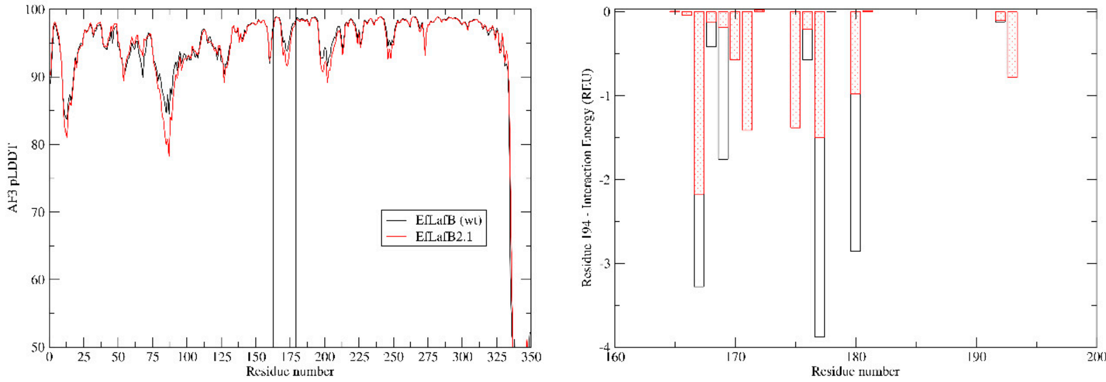

Figure S4. (Left) AlphaFold3 pLDDT confidence scores per residue for wild-type EfLafB (black) and mutant EfLafB2.1 (red). The shaded region between the vertical lines indicates residues 165-180, encompassing the environment surrounding residue R/W193. (Right) Interaction energies between residue 193 and surrounding residues in wild-type EfLafB (black) and mutant EfLafB2.1 (red). Prior to energy calculations, models were relaxed using the Rosetta FastRelax protocol
